# Shiny binary modeling platform: a reproducibility-oriented software platform for structured binary modeling with integrated interpretation, validation support, model comparison, and archive export

**DOI:** 10.3389/fphar.2026.1905644

**Published:** 2026-09-03

**Authors:** Haikun Du, Xiaozhe Huang, Tianxiang Liu, Arzuye Dolkun

**Affiliations:** School of Pharmacy, Xinjiang Medical University, Urumqi, China

**Keywords:** archive export, model comparison, reproducible workflow, SHAP, Shiny, structured binary modeling

## Abstract

**Background:**

Structured binary modeling is often implemented through scattered scripts and temporary files, leaving preprocessing recipes, benchmarks, deployed models, interpretation outputs, and archive materials difficult to trace. This limits practical reproducibility and later reuse.

**Results:**

We developed Shiny Binary Modeling Platform, an R/Shiny application that links data setup, preprocessing retention, candidate-model benchmarking, final model deployment, SHAP-based interpretation, external-validation support, model comparison, and archive export in one project workflow. In a demonstration using 6383 traditional Chinese medicine-derived compound records, a source-derived pure-toxic-source label, and 12 ADME/physicochemical descriptors, the platform evaluated 113 candidate model specifications under stratified 10-fold cross-validation. Majority-class downsampling was applied only within training folds; assessment folds retained the original imbalanced distribution. The descriptor set was used to exercise the software workflow and should not be interpreted as a comprehensive endpoint-specific toxicity-QSAR feature set. No independent external validation dataset was used, so reported performance represents internal cross-validation only. Stepglm [both]+RF—a two-stage specification using both-direction stepwise logistic-regression screening followed by random forest—served as the primary performance-summary model for ROC/PR reporting and structured export. With pure_toxic_label = 1 as the toxic/positive class, its merged out-of-fold PR-AUC was 0.2469; at threshold 0.5, toxic-class precision/PPV, recall/sensitivity, F1, and NPV were 0.2288, 0.4091, 0.2935, and 0.9509. Archived global and local SHAP outputs were re-audited separately: the global outputs shown in were traced to the retained Lasso + RF historical reference project, whereas the local waterfall output shown in was traced to the Stepglm [both]+RF primary performance-summary model.

**Conclusion:**

This is a software article rather than a final endpoint-specific prediction study. The current use case demonstrates integrated benchmarking, deployment, internal OOF reporting, audited SHAP-output handling, model comparison, and reproducible export. External-validation support and standalone prediction-app export are implemented capabilities, but the single-dataset demonstration does not support independent external-performance, deployment-readiness, or competitive toxicity-QSAR claims.

## 1 Introduction

Structured binary modeling is widely used in screening, prioritization, and risk stratification tasks, but the surrounding analytical workflow is often under-described. In many practical settings, data import, preprocessing, candidate-model benchmarking, final deployment, interpretation, validation, and export are distributed across multiple scripts or notebooks. Core analytical objects may therefore be retained inconsistently, and a completed analysis can be difficult to reconstruct later.

Recent work in predictive toxicology has shown sustained growth in the use of machine learning for endpoint classification and ranking tasks (Zulkifli et al., 2023; Cavasotto and Scardino, 2022; Wang et al., 2021; Mayr et al., 2016; Seal et al., 2025). Endpoint-oriented toxicity studies commonly rely on experimentally defined outcomes and richer structural representations, such as molecular fingerprints, extended molecular-descriptor panels, or structural alerts (Zulkifli et al., 2023; Cavasotto and Scardino, 2022; Wang et al., 2021; Mayr et al., 2016; Seal et al., 2025). Explainability approaches such as SHAP have also become increasingly common for examining how model predictions are formed (Lundberg and Lee, 2017; Konig and Vellido, 2024). However, these advances do not by themselves resolve workflow fragmentation. In many studies, model training, interpretation, validation, and reporting are still implemented as partially disconnected steps, leaving the overall analytical chain difficult to trace or reproduce in a standardized way (Abou Hajal and Al Meslamani, 2024; Wheeler et al., 2023; Wilson et al., 2014; Sandve et al., 2013).

We therefore addressed a software gap rather than proposing a new learning algorithm or a new endpoint-specific toxicity predictor. Shiny Binary Modeling Platform was developed as an interactive R/Shiny application that consolidates candidate-model benchmarking, deployment, SHAP-based interpretation, external-validation support, model comparison, and archive export within one reproducibility-oriented workflow. A classification task based on traditional Chinese medicine-derived compounds is used only as a demonstration case to show how the platform operates in a realistic setting, not as a toxicity-QSAR benchmark.

## 2 Materials and methods

### 2.1 Design objective

The primary design objective was to reduce workflow fragmentation in structured binary modeling. Rather than optimizing around one learner family or one toxicity endpoint, the software was designed to support a reusable analysis chain in which preprocessing, benchmarking, deployment, interpretation, validation, comparison, and export remain connected within one project object.

### 2.2 Software environment and workflow architecture

The platform was implemented in R with Shiny and shinydashboard as the interactive interface framework (Chang et al., 2016). The current implementation is organized into six sequential modules: data setup and preprocessing, candidate-model evaluation, final model deployment, SHAP-based interpretation, external-validation support, and model comparison. These modules are designed to operate on the same evolving project object so that later stages can directly reuse earlier analytical outputs without rebuilding intermediate objects outside the application.

### 2.3 Modeling engine and candidate-model construction

The modeling engine was independently implemented by the authors within this project as learner-specific R wrappers and a unified dispatcher; it was not adapted from Mime, and no Mime source code was copied, imported, or called. The general strategy of evaluating multiple single-learner and two-stage learner combinations was informed by prior multi-learner benchmarking frameworks such as Mime (Liu et al., 2024), which is cited for methodological context. Each learner is fitted through its standard R package: glmnet for Lasso, Ridge, and Elastic Net; stats for stepwise logistic regression (Stepglm); randomForestSRC for random forest; e1071 and caret for support vector machines, naive Bayes, and linear discriminant analysis; mboost for glmBoost; plsRglm for partial least-squares logistic regression; gbm for gradient boosting; and xgboost for XGBoost. The MIT license supplied with the project applies to the authors' application and wrapper code; third-party R packages remain governed by their own licenses.

For the demonstration task, the candidate list comprised 113 fixed model specifications. These included 22 single-learner specifications and two-stage combinations in which one of five first-stage screening methods (Lasso, RF, Stepglm [backward], Stepglm [both], or glmBoost) selected variables before a downstream learner was fitted. This candidate list was used to exercise the platform benchmarking, deployment, interpretation-output handling, model comparison, and export workflow; it should not be interpreted as a newly proposed learning algorithm. The platform-specific contribution is the Shiny workflow layer, retained project-object structure, unified candidate-model dispatch, model deployment, SHAP-linked interpretation workflow, external-validation interface, model-comparison interface, and archive export. The external-validation interface is described as software support rather than as evidence of external validation in the current use case. Here, Stepglm [both]+RF denotes a two-stage specification in which both-direction stepwise logistic-regression screening is followed by random forest classification.

### 2.4 Retained project objects and archive strategy

One of the main software design choices was to preserve analytical objects that are often lost in a scattered script workflow. The platform retains the preprocessing recipe, candidate-model summaries, fold-level prediction outputs, deployed model objects, SHAP matrices, feature-importance summaries, comparison metrics, and archive-ready exports. At the end of an analysis session, it can also export a structured project bundle containing tables, figures, model-related project materials, and a standalone prediction app generated from the deployed model and associated preprocessing objects.

During revision, we expanded the software documentation and reproducibility materials. The revised code package includes architecture and extensibility documentation, a reproducibility guide, example-workflow and expected-output guidance, installation instructions, dependency records, and a project-local renv generation script. We additionally performed an environment audit on the author’s Windows 11 × 64 computer using R 4.5.2. The submitted app.R passed a non-interactive source smoke test, and all 38 packages explicitly loaded by the application were available. Exact declared-package versions and full session information are included in the revised software archive. A validated renv.lock is not claimed because renv was not available in the tested environment and a clean-machine renv::restore () test could not be completed. The supplied version inventory and session record therefore provide an equivalent audit record for this revision but not a fully restore-tested environment lock.

### 2.5 Demonstration dataset and task configuration

To demonstrate the software in a realistic scenario, the platform was applied to a structured binary classification task based on 6383 traditional Chinese medicine-derived compound-level records. The outcome pure_toxic_label is database-derived through a deterministic source-annotation rule; it is neither experimentally measured at compound level nor generated by a prediction model. The frozen table retained source_herbs_all, source_herbs_toxic, total_herbs, toxic_herbs, and toxicity_prob, where toxicity_prob = toxic_herbs/total_herbs. A compound was assigned pure_toxic_label = 1 only at the prespecified threshold toxicity_prob = 1, meaning that all retained source herbs were annotated as toxic-source herbs (toxic_herbs = total_herbs). Compounds with no toxic-source herb or with mixed toxic and non-toxic source herbs were assigned pure_toxic_label = 0. This label was used to create an imbalanced software-demonstration task and should not be interpreted as a validated experimental toxicological endpoint.

The frozen table contained 6383 unique mol_id records, no duplicated mol_id values, no missing pure_toxic_label values, 462 positives, and 5921 negatives, corresponding to a positive proportion of 7.24%. A rule audit performed during revision found no mismatch between the retained pure_toxic_label values and the source-derived rule; the data dictionary and record-level rule audit are provided in Additional file 12. Twelve ADME/physicochemical descriptors were used as predictors: ob, mw, alogp, caco2, bbb, halflife, hdon, hacc, dl, fasa, rbn, and tpsa. The descriptor naming and organization are consistent with the ADME/physicochemical field system commonly represented in TCMSP (Ru et al., 2014). These compact descriptors were sufficient for exercising data setup, benchmarking, deployment, interpretation, and export modules, but they do not constitute a rich endpoint-specific toxicity-QSAR feature set and do not include molecular fingerprints, expanded structural descriptors, or structural-alert features. Because this was a computational software demonstration rather than an experimental detection or analytical quantification assay, no limit of detection (LOD) or limit of quantification (LOQ) was estimated. The platform operates on user-supplied tabular predictors and binary labels; in the present example, the relevant limits concern label provenance, descriptor scope, class imbalance, and validation design rather than compound-level detection or concentration quantification.

### 2.6 Benchmarking, deployment, and interpretation configuration

The demonstration task was evaluated under stratified 10-fold cross-validation. For each split, the assessment fold was separated before preprocessing or resampling. The preprocessing recipe was fitted on the analysis portion and then applied to the held-out assessment fold. Majority-class downsampling to a 1:1 ratio was used only within each analysis portion; assessment folds retained the original class distribution. The 113 candidate specifications were evaluated using AUC, PR-AUC, Brier score, and positive-class threshold metrics calculated from untouched OOF predictions. Calibration was summarized using mean predicted probability versus observed prevalence, calibration intercept and slope, and 10-bin expected calibration error. No independent external dataset satisfying the frozen model’s compatibility requirements was available. A valid external dataset would require an independently assembled population, the same predictor definitions and units (or a prespecified reproducible mapping), and a comparable outcome provenance and positive-class definition; an unrelated public binary-classification dataset would not constitute external validation. We separately performed an interface-operability smoke test: the frozen Stepglm [both]+RF project generated 6383 finite probabilities (range 0–0.9599) when its retained preprocessing recipe and model were applied to compatible records. This test demonstrates software execution only and is not used as independent external-performance evidence. All reported performance estimates remain internal cross-validation results. The archived global SHAP outputs correspond to the retained Lasso + RF historical reference project, whereas the local SHAP waterfall output corresponds to the Stepglm [both]+RF performance-summary model.

## 3 Results

### 3.1 Workflow outputs from the demonstration case

The software completed the main single-dataset workflow chain within one interface: data setup, preprocessing retention, candidate-model benchmarking, deployment, interpretation-output handling, model comparison, and archive export. The demonstration run produced benchmark summaries, fold-level predictions, a deployed model object, audited model-specific SHAP outputs, model-comparison outputs, and archive-ready exports without moving the analysis into separate external scripts. The external-validation interface was implemented as part of the platform workflow, but it was not used to claim external performance in this single-dataset demonstration.

To distinguish software-supported functionality from evidence generated in the current use case, we classified the workflow outputs explicitly during revision. The present demonstration generated evidence for data setup, preprocessing retention, candidate-model benchmarking, internal OOF performance reporting, deployment of a selected performance-summary model, audited model-specific SHAP outputs, model-comparison outputs, and structured archive export. By contrast, the external-validation module, the interface for applying a frozen model to a user-supplied compatible dataset, and standalone prediction-app export are described as implemented software capabilities; they were not used in this single-dataset demonstration to claim independent external performance, deployment readiness, or cross-dataset generalization.

### 3.2 Candidate-model benchmarking

**FIGURE 1:**
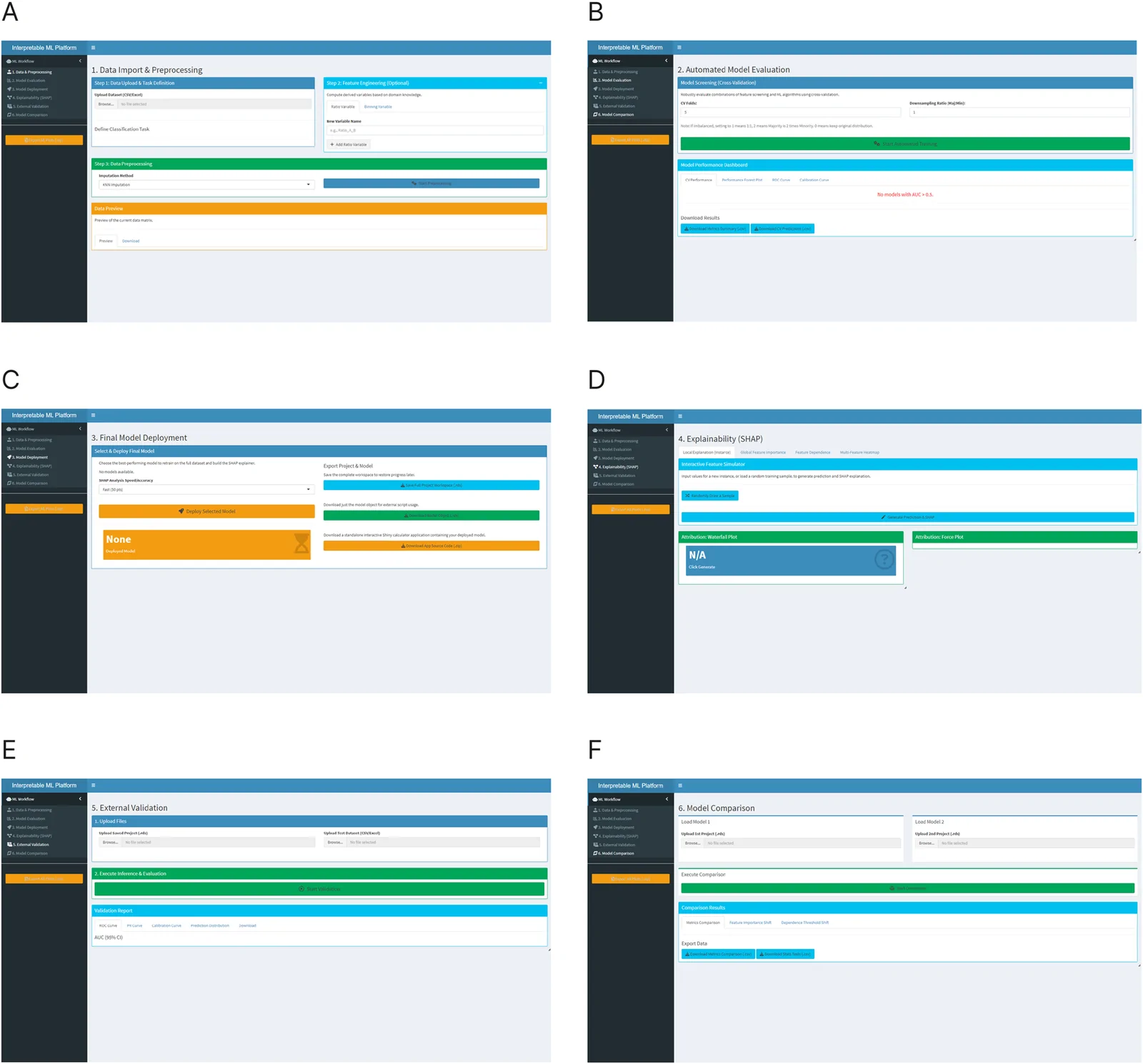
Representative interface screenshots of the Shiny Binary Modeling Platform. **(A)** Data setup and preprocessing, **(B)** Candidate-model evaluation, **(C)** Final model deployment, **(D)** SHAP interpretation, (E) External-validation support, **(F)** Model comparison.

The Shiny Binary Modeling Platform provides a unified graphical interface for structured binary modeling, covering data import, preprocessing, model evaluation, final deployment, SHAP-based interpretation, external-validation support, model comparison, and result export. Figure 1 summarizes dataset upload, task definition, optional feature engineering, imputation-based preprocessing, cross-validation, training-fold-only downsampling, automated model training, performance visualization, deployment, project/model export, local and global interpretation, compatible-data prediction, and side-by-side model comparison. Evidence generated in the present study comprises internal OOF performance, retained project objects, audited model-specific SHAP outputs, model-comparison outputs, archive export, and a compatible-data interface smoke test. The latter verifies execution but is not an independent external validation analysis. Accordingly, the manuscript uses “external-validation support” or “external-validation interface” and makes no external-generalization claim.

Platform interface and workflow outputs are summarized in Figure 1.

### 3.3 Deployed demonstration model

After benchmarking, Stepglm [both]+RF was selected as the primary performance-summary model. The corresponding selected descriptor set contained eight descriptors: hdon, dl, alogp, ob, fasa, halflife, hacc, and rbn. Using the combined OOF predictions from untouched assessment folds, Stepglm [both]+RF achieved a ROC-AUC of 0.7439, a PR-AUC of 0.2469, and a Brier score of 0.1230. Across the 10 assessment folds, the fold-level PR-AUC mean±SD was 0.2677±0.0495 and the fold-level Brier score mean±SD was 0.1230±0.0040. The combined OOF prediction set retained all 6383 original records, including 462 positive and 5921 negative observations. At a fixed 0.5 threshold and with pure_toxic_label = 1 treated as the toxic/positive event class, the confusion matrix was TP = 189, FP = 637, TN = 5284, and FN = 273; the corresponding toxic-class precision/PPV, recall/sensitivity, F1, and NPV were 0.2288, 0.4091, 0.2935, and 0.9509, respectively. A threshold selected to maximize positive-class F1 was 0.4671, yielding precision/PPV = 0.2164, recall/sensitivity = 0.4913, and F1 = 0.3005. Calibration summaries from the combined OOF predictions showed that the mean predicted probability was 0.2945 compared with an observed positive prevalence of 0.0724, indicating probability overestimation in this demonstration. The calibration intercept was −1.8297, the calibration slope was 1.0997, and the 10-bin expected calibration error was 0.2221.

The relevance of this result within the present article lies less in claiming a definitive endpoint model and more in showing that the benchmarking and reporting workflow operated successfully on a realistic imbalanced dataset. Given the source-derived label, compact descriptor set, and lack of independent external validation, these metrics should be read as workflow audit outputs rather than as endpoint-specific toxicity-QSAR performance estimates. The 113-combination search did not provide a practically meaningful improvement over a single RF model in this demonstration: Stepglm [both]+RF and RF had very similar merged OOF ROC-AUC (0.7439 versus 0.7421), PR-AUC (0.2469 versus 0.2466), and Brier score (0.1230 versus 0.1231). RF also showed similar fold-level PR-AUC mean±SD (0.2674±0.0439), fold-level Brier score mean±SD (0.1231±0.0052), and calibration summaries (intercept = −1.8213, slope = 1.1184, 10-bin expected calibration error = 0.2234). Stepglm [both]+RF should therefore be read as a representative primary performance-summary model for exercising the benchmarking workflow, not as evidence that this candidate structure is broadly superior. The PR-AUC, Brier, and calibration audit is provided in Additional file 15.

**FIGURE 2:**
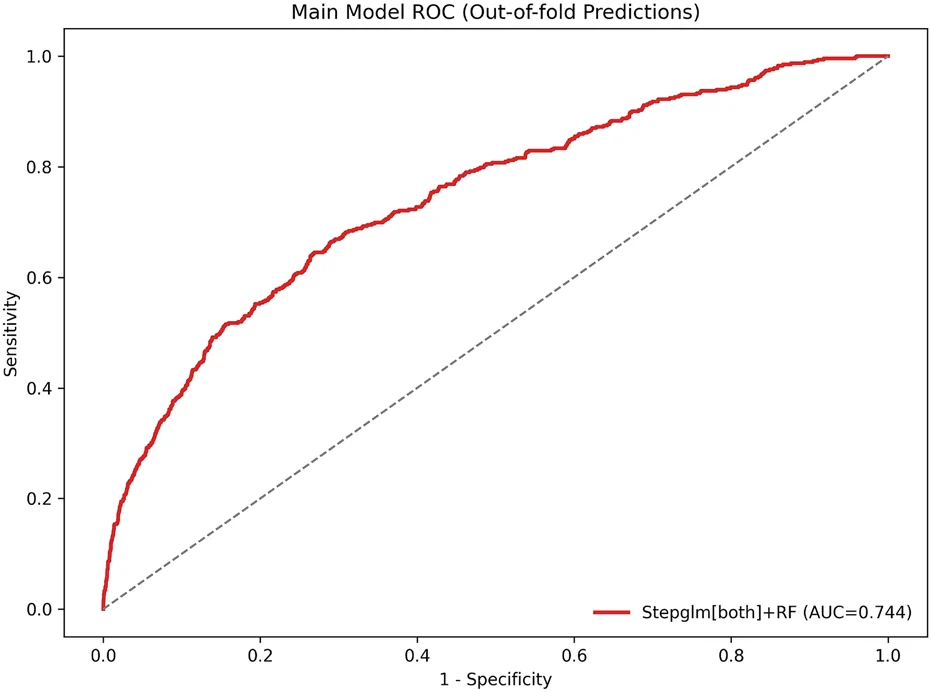
ROC output for the Stepglm [both]+RF primary performance-summary model.

The ROC output for the primary performance-summary model is shown in Figure 2; the PR curve is retained as a supplementary performance display.

### 3.4 Global and local SHAP outputs

**FIGURE 3:**
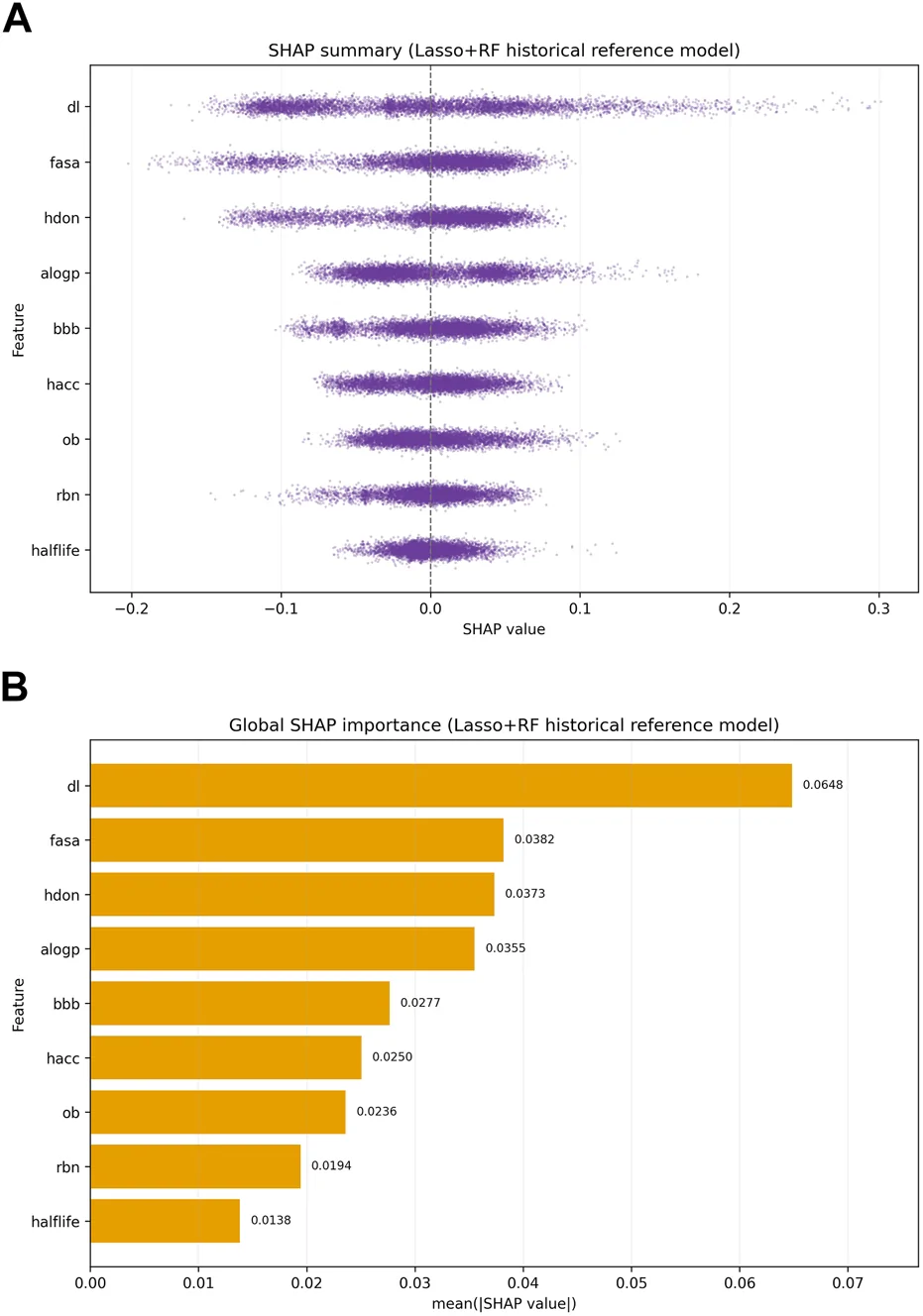
Global SHAP outputs generated from the retained Lasso + RF historical reference project. The figure is included solely to demonstrate the platform’s audited SHAP export workflow; it is not an interpretation of the Stepglm [both]+RF primary performance-summary model.

**FIGURE 4:**
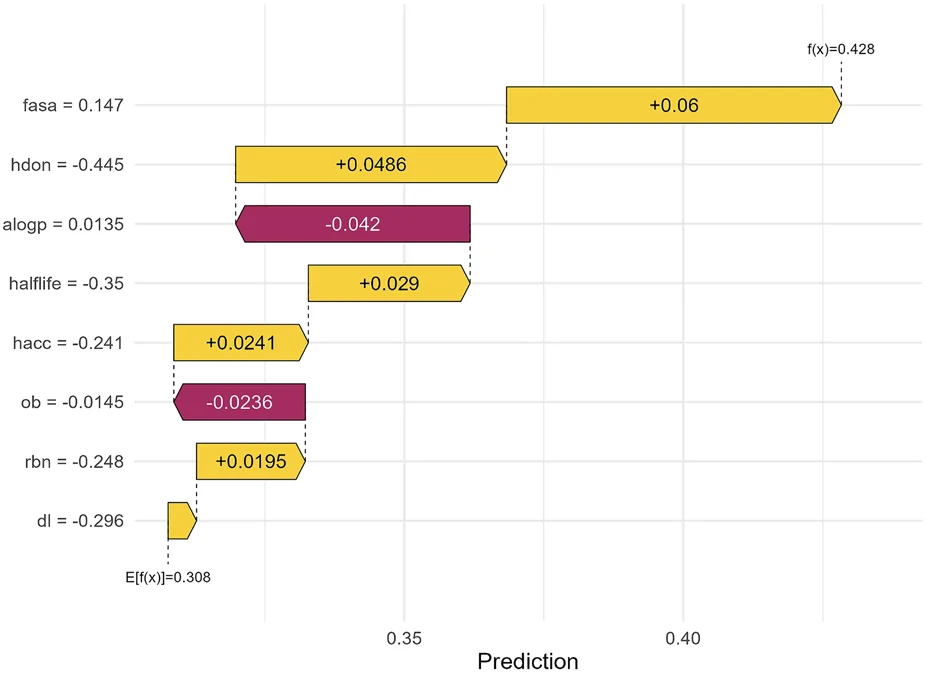
Local SHAP waterfall output for a representative sample from the Stepglm [both]+RF primary performance-summary model. The figure is included to demonstrate the platform’s audited local-SHAP export workflow.

During revision, all SHAP artifacts were re-audited against the retained feature tables and model-specific project objects. The global SHAP table and Figure 3 were confirmed to correspond to the Lasso + RF historical reference model, whose retained features were ob, alogp, bbb, halflife, hdon, hacc, dl, fasa, and rbn. In this audited global SHAP summary, dl, fasa, hdon, alogp, and bbb had the largest mean absolute SHAP values, with hacc, ob, rbn, and halflife also retained in the model. By contrast, the local SHAP waterfall plot in Figure 4 contains the eight descriptors retained by the Stepglm [both]+RF primary performance-summary model: hdon, dl, alogp, ob, fasa, halflife, hacc, and rbn. Figure 4 is therefore attributed to the Stepglm [both]+RF model. Each SHAP output is labelled with its corresponding source model.

Within the logic of this manuscript, these outputs are presented as software demonstrations of global and local explanation capability rather than as biological mechanism claims. They show that the corresponding model-specific project objects can be directly connected to interpretation outputs and then included in the same structured export pathway.

The audited global SHAP outputs from the Lasso + RF historical reference model and the audited local SHAP output from the Stepglm [both]+RF primary performance-summary model are shown in Figures 3, 4, respectively.

### 3.5 Workflow-level comparison with a conventional scattered R script workflow

For a software article, the more defensible comparison target is workflow organization rather than a single model score.

A workflow-level comparison with a conventional scattered R script workflow is provided in Table 1.

**TABLE 1:** Workflow-level comparison with a conventional scattered R script workflow.

Dimension	Conventional scattered R script workflow	Current platform
Task entry	Handled across multiple scripts or notebooks	Defined in one interface
Retained preprocessing objects	Manually saved and easy to lose	Recipe and project object retained together
Candidate-model benchmarking	Often manually summarized	Unified benchmarking within one session
Freezing of deployed models	Often requires extra scripts	Generated directly inside the platform
Global/local interpretation	Usually a post-processing add-on	Linked directly to the deployed model
External-validation support	Commonly implemented separately	Dedicated in-platform support module
Model comparison	Often manual and fragmented	Structured comparison inside one workflow
Archive export	Commonly assembled by hand	One-click structured project export
Quantitative benchmark context	Requires a prespecified, hardware-matched protocol	One tested complete run: 6383 records, 12 descriptors, 113 specifications, 10 folds; 518.509 s, 1130/1130 successful fits, observed peak working set approximately 2.08 GiB

Overall, the main gain of the platform does not lie in a claim of competitive toxicology prediction performance or universally superior predictive performance. It lies in bringing task definition, candidate-model benchmarking, deployment, interpretation, validation support, comparison, and archive export into one retained project structure and one export chain.

## 4 Discussion

### 4.1 Main software contribution

The present work contributes a software workflow rather than a new endpoint-specific prediction method or a new toxicity-QSAR model. Its main value lies in integrating data setup, benchmarking, deployment, interpretation, validation support, comparison, and archive export into one reproducibility-oriented interface. In practice, these steps are often completed through a scattered script workflow that may be sufficient for a single analysis but is less convenient for preserving a full decision trail and the associated analytical objects (Abou Hajal and Al Meslamani, 2024; Wheeler et al., 2023; Wilson et al., 2014; Sandve et al., 2013).

### 4.2 Why the single use case still matters

The 6383-compound demonstration case remains useful because it shows the platform operating under a realistic structured binary task with class imbalance, repeated candidate-model benchmarking, deployment of a selected model, and SHAP-based interpretation. The case is therefore presented as a use-case demonstration for software feasibility, not as the basis for broad endpoint-level generalization or competitive toxicity-QSAR claims.

### 4.3 Relationship to related software and predictive studies

Existing studies and tools address endpoint-specific toxicity prediction, ADMET evaluation, dose-response analysis, reproducible computational workflow design, or toxicology-oriented workflow support using molecular descriptors, structural representations, and explainability tools (Zulkifli et al., 2023; Cavasotto and Scardino, 2022; Wang et al., 2021; Mayr et al., 2016; Seal et al., 2025; Lundberg and Lee, 2017; Konig and Vellido, 2024; Abou Hajal and Al Meslamani, 2024; Wheeler et al., 2023; Wilson et al., 2014; Sandve et al., 2013; Chang et al., 2016; Liu et al., 2024; Pu et al., 2019; de Sa et al., 2022; Yang et al., 2019; Gu et al., 2024; Xiong et al., 2021; Jaganathan et al., 2022; Jiang et al., 2024; Goel et al., 2025). Within BMC Pharmacology and Toxicology, related computational work has included eToxPred for machine-learning toxicity estimation (Pu et al., 2019), *in silico* screening of essential medicines for drug-induced toxicity risk (Chikowe et al., 2021), and computational ADMET/toxicity-risk assessment of repurposed approved drugs (Aminpour et al., 2021). These studies provide related context rather than direct performance benchmarks for the present software article. At the platform level, ToxicR (Wheeler et al., 2023), admetSAR 2.0 (Yang et al., 2019), admetSAR3.0 (Gu et al., 2024), and ADMETlab 2.0 (Xiong et al., 2021) represent common local or web-based implementations. More recent work also includes a broad review of structure-based toxicity prediction (Seal et al., 2025), workflow-oriented toxicological software such as ToxDAR (Jiang et al., 2024), and online toxicity prediction tools such as ToxinPredictor (Goel et al., 2025). Table 2 therefore compares the present platform with related platforms at the level of publicly described functions and delivery form.

**TABLE 2:** Functional comparison with related toxicology and ADMET software platforms.

Platform	Delivery	Primary analytical scope	User-data model benchmarking	Interpretation and validation support	Export or retained project outputs
ToxicR (Wheeler et al., 2023)	Local R platform	Computational toxicology and dose-response analysis	Dose-response model analysis rather than candidate-learner benchmarking for user-defined binary outcomes	Dose-response reporting and visualization; no deployed binary-model SHAP or external-validation workflow described	R-based analysis outputs
admetSAR 2.0 (Yang et al., 2019)	Web service	Chemical ADMET prediction and optimization	Online endpoint prediction rather than local multi-model benchmarking	Online ADMET prediction results	Online result return
admetSAR3.0 (Gu et al., 2024)	Web platform	ADMET exploration, prediction, and optimization	Multi-property online prediction rather than local multi-model benchmarking	Online ADMET profile exploration and prediction results	Online result return
ADMETlab 2.0 (Xiong et al., 2021)	Web platform	Multi-endpoint ADMET prediction	Endpoint-oriented online evaluation rather than local multi-model benchmarking	Online ADMET profile results	Online result return
ToxDAR (Jiang et al., 2024)	Workflow software	Toxicologically relevant proteomic and transcriptomic data analysis	Omics/toxicology workflow rather than small-molecule binary-classifier benchmarking	Mechanism-oriented omics analysis	Workflow output files
ToxinPredictor (Goel et al., 2025)	Online prediction tool	Molecular toxicity prediction	Endpoint prediction rather than local multi-model benchmarking	Toxicity prediction outputs	Online result return
Shiny Binary Modeling Platform (this study)	Local R/Shiny platform	Unified workflow for structured binary modeling	113 candidate model specifications evaluated in one session	Global/local SHAP workflow, external-validation support, and side-by-side model comparison	Structured project bundle and standalone prediction app

A functional comparison with related toxicology and ADMET software platforms is provided in Table 2. It uses objective capability categories to clarify scope and workflow coverage rather than claim performance superiority. We reran the complete demonstrated workload on the author’s Windows 11 × 64 computer using R 4.5.2: 6383 records, 12 descriptors, 113 candidate specifications, and stratified 10-fold cross-validation. The single complete run required 518.509 s (8.64 min), completed all 1130 model-fold fits without a recorded error, and reached an observed peak R-process working set of approximately 2.08 GiB. Runtime and memory measurements are summarized in Table 1. These measurements demonstrate local operability at the reported workload; they are not a multi-machine scalability study and do not support a claim of speed superiority over other tools. Usability comparisons remain limited to observable workflow integration and retained project outputs.

From this perspective, the present platform is not intended to replace existing online prediction services or compete with endpoint-specialized toxicology-QSAR systems. Its contribution is at the local project level, where workflow integration, retained analytical objects, and structured export are emphasized.

### 4.4 Current limits and next steps

The current study has several clear limits. First, the fully described use case is a single demonstration dataset, and the compatible-data interface smoke test is not independent external validation. Second, the benchmark results do not justify broad superiority claims: Stepglm [both]+RF and plain RF had nearly identical archived OOF discrimination, PR-AUC, and Brier score, and the 113-specification search should be interpreted as a workflow demonstration. Third, the global SHAP outputs in Figure 3 originate from the retained Lasso + RF historical reference model, whereas the local SHAP output in Figure 4 originates from the Stepglm [both]+RF primary performance-summary model; both are presented to demonstrate audited output handling rather than biological mechanism. Fourth, pure_toxic_label is a database-derived source-annotation label, not experimentally confirmed compound toxicity. Fifth, the 12 ADME/physicochemical descriptors are a compact workflow-demonstration set and omit molecular fingerprints, expanded structural descriptors, structural alerts, and other richer endpoint-specific representations. Sixth, positive-class performance and calibration remained modest and should not be interpreted as deployment-ready toxic-compound identification. Seventh, the measured runtime (518.509 s; observed peak working set approximately 2.08 GiB) comes from one complete rerun on one Windows 11 × 64 computer and is not a multi-machine scalability comparison. Eighth, exact package versions, session information, seed-control documentation, example data, expected outputs, and smoke-test records are supplied, but a clean-machine renv::restore () test and tested Docker image were not completed. Independent external validation, richer endpoint-specific feature engineering, repeated hardware benchmarks, broader multi-task demonstrations, and formal environment restoration remain future work.

## 5 Conclusion

This work presents a Shiny-based software platform for structured binary modeling with integrated interpretation, validation support, model comparison, and archive export. In the current demonstration case, the platform successfully organized candidate-model benchmarking, deployment of a selected performance-summary model, internal OOF reporting, audited interpretation-output handling, model-comparison outputs, and retained-object export within one reproducibility-oriented workflow. The current evidence supports platform feasibility and workflow integration rather than broad predictive claims across endpoints or datasets, and it should not be interpreted as evidence of competitive toxicity-QSAR performance, independent external generalization, or deployment-ready standalone prediction. The software is publicly accessible via the GitHub repository of the Shiny Binary Modeling Platform (Shiny Binary Modeling Platform, 2025), as well as the tagged reviewed release version v0.1.0 (Shiny Binary Modeling Platform v0, 2026).

## Supplementary material

SUPPLEMENTARY DATA SHEET 1 Supplementary figures and legends.

SUPPLEMENTARY DATA SHEET 2–10 Machine-readable workflow outputs.

SUPPLEMENTARY DATA SHEET 11 Frozen review release and revised software documentation.

SUPPLEMENTARY SUPPLEMENTARY TABLES 1–5 Outcome—label audit, positive-class performance audit, SHAP consistency audit, PR-AUC/Brier/calibration audit, and software reproducibility audit, respectively.
