# Supplementary material for: Shiny binary modeling platform: a reproducibility-oriented software platform for structured binary modeling with integrated interpretation, validation support, model comparison, and archive export
Source: Front Pharmacol. 2026 Sep 3;17:1905644. doi: 10.3389/fphar.2026.1905644 (PMC13582506; doi:10.3389/fphar.2026.1905644)
Supplement: Supplementary file 1 [file DataSheet1.pdf]

Additional file 1. Supplementary Information

For the manuscript:

Shiny Binary Modeling Platform: a reproducible software platform for structured binary modeling with integrated interpretation, validation, model comparison, and archive export

This supplementary file is aligned with the frozen main-text version centered on the 2026-03-06 Stepglm[both]+RF deployment. Alternative exported models, including Lasso+RF, RF, and SVM, are retained only as supplementary comparators for version tracing and consistency checks.

Supplementary figure numbering starts from Figure S2 to remain consistent with the numbering already cited in the frozen main-text version.

Supplementary Figures

Figure S2. Calibration curve of the deployed Stepglm[both]+RF model

This figure supplements the main-text performance results by showing the calibration pattern of the deployed model. Because the current task is highly imbalanced, the calibration result is presented as supportive evidence rather than as a main-text core figure.

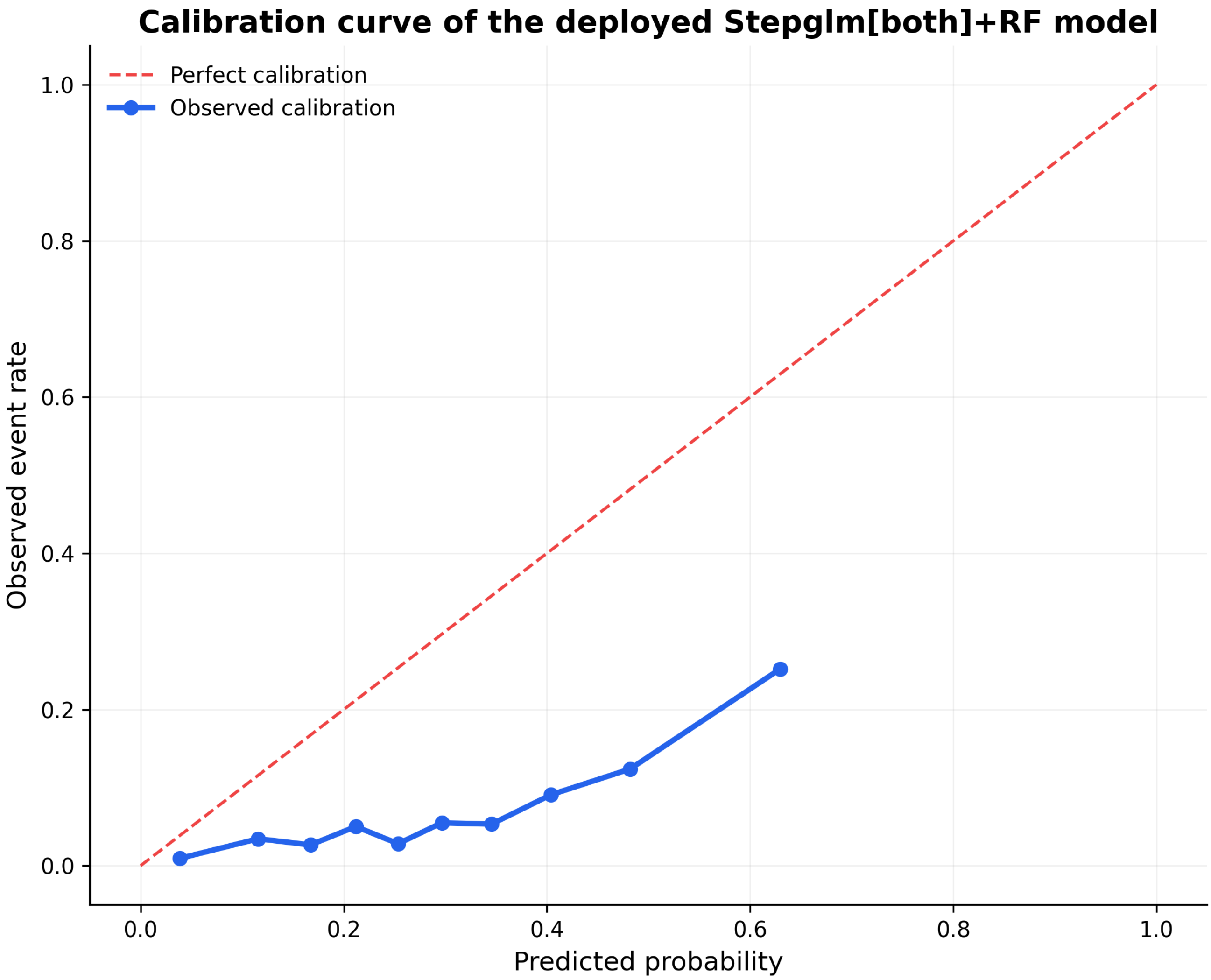

Figure S3. Global SHAP bar plot of the deployed model

This figure complements the main-text SHAP beeswarm plot by reporting the ranking of mean absolute SHAP values for the retained features.

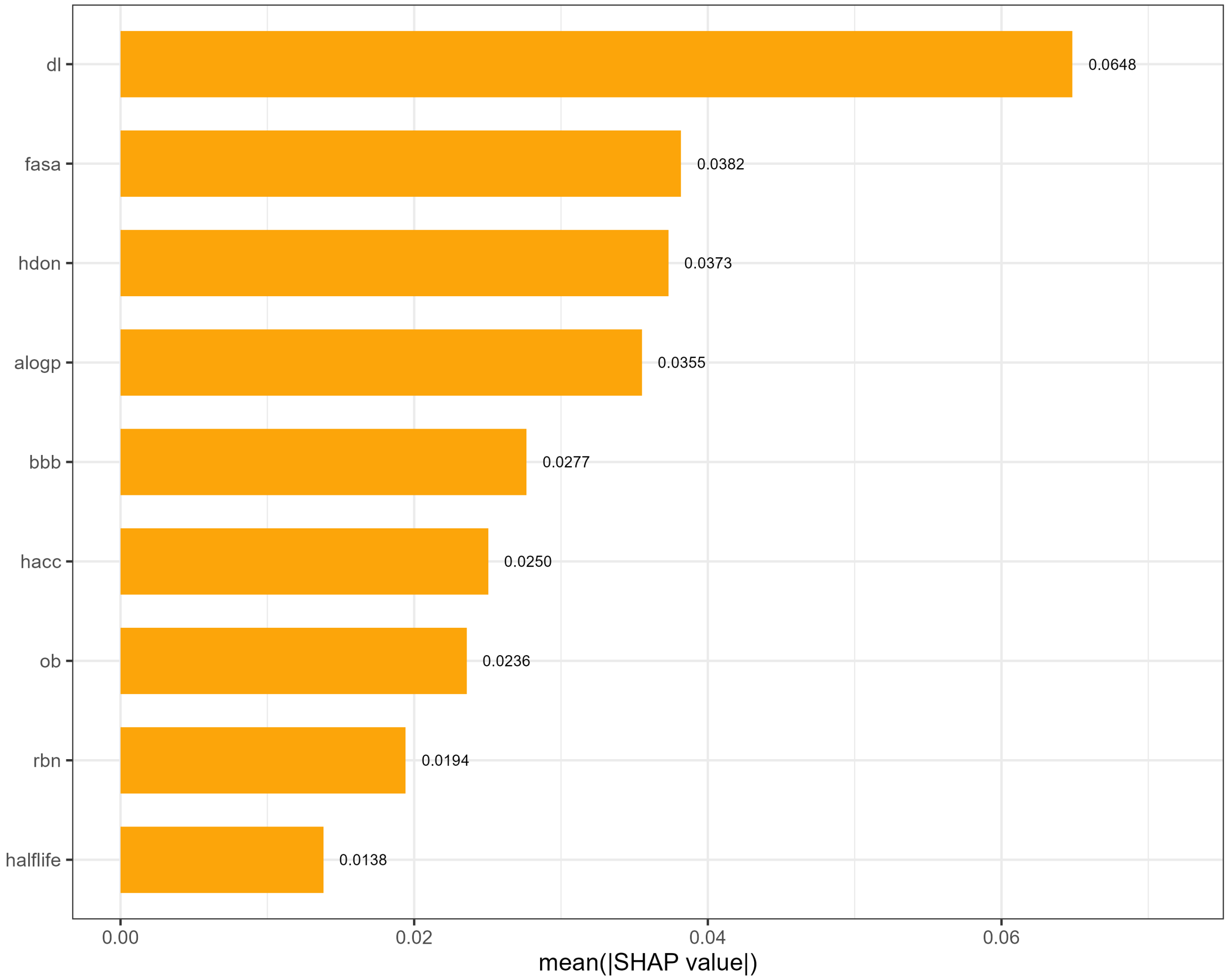

Figure S4. Local SHAP force plot for the representative sample

This figure supplements the local SHAP waterfall plot shown in the main text and illustrates how the final prediction for the representative sample is jointly shaped by multiple retained descriptors.

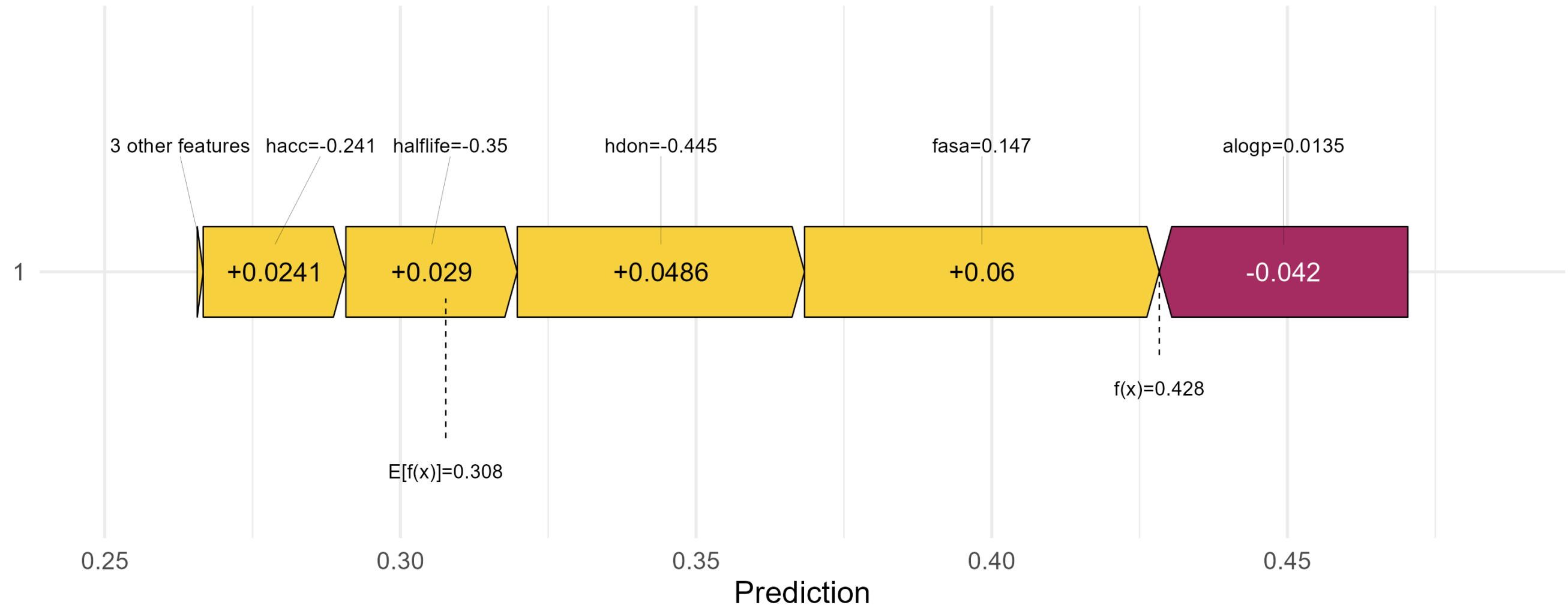

Figure S5. SHAP dependence plot for hdon

This figure shows the dependence pattern between hdon values and their associated SHAP contributions in the deployed model.

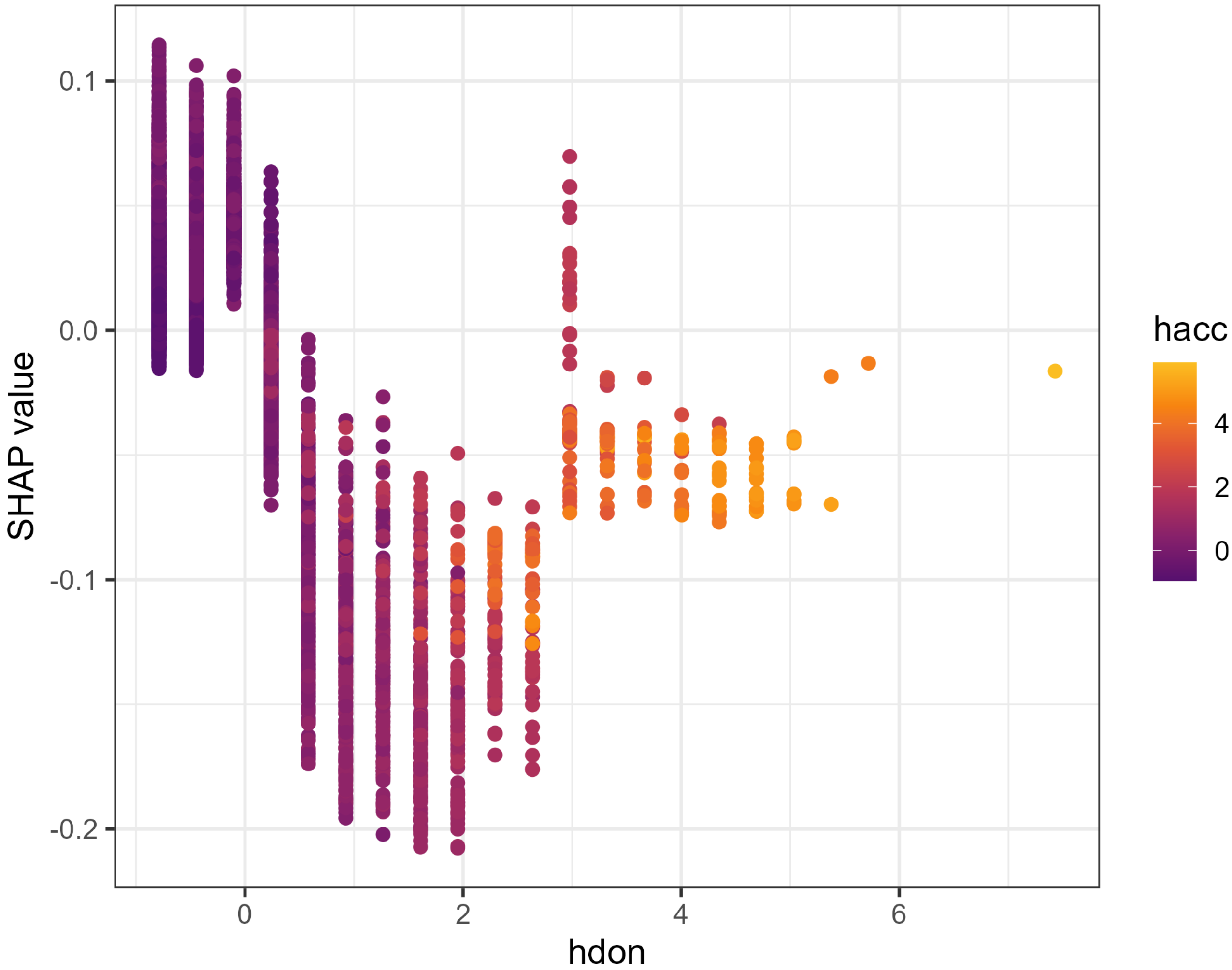

Figure S6. SHAP dependence plot for fasa

This figure shows the non-linear contribution pattern of fasa across the observed value range.

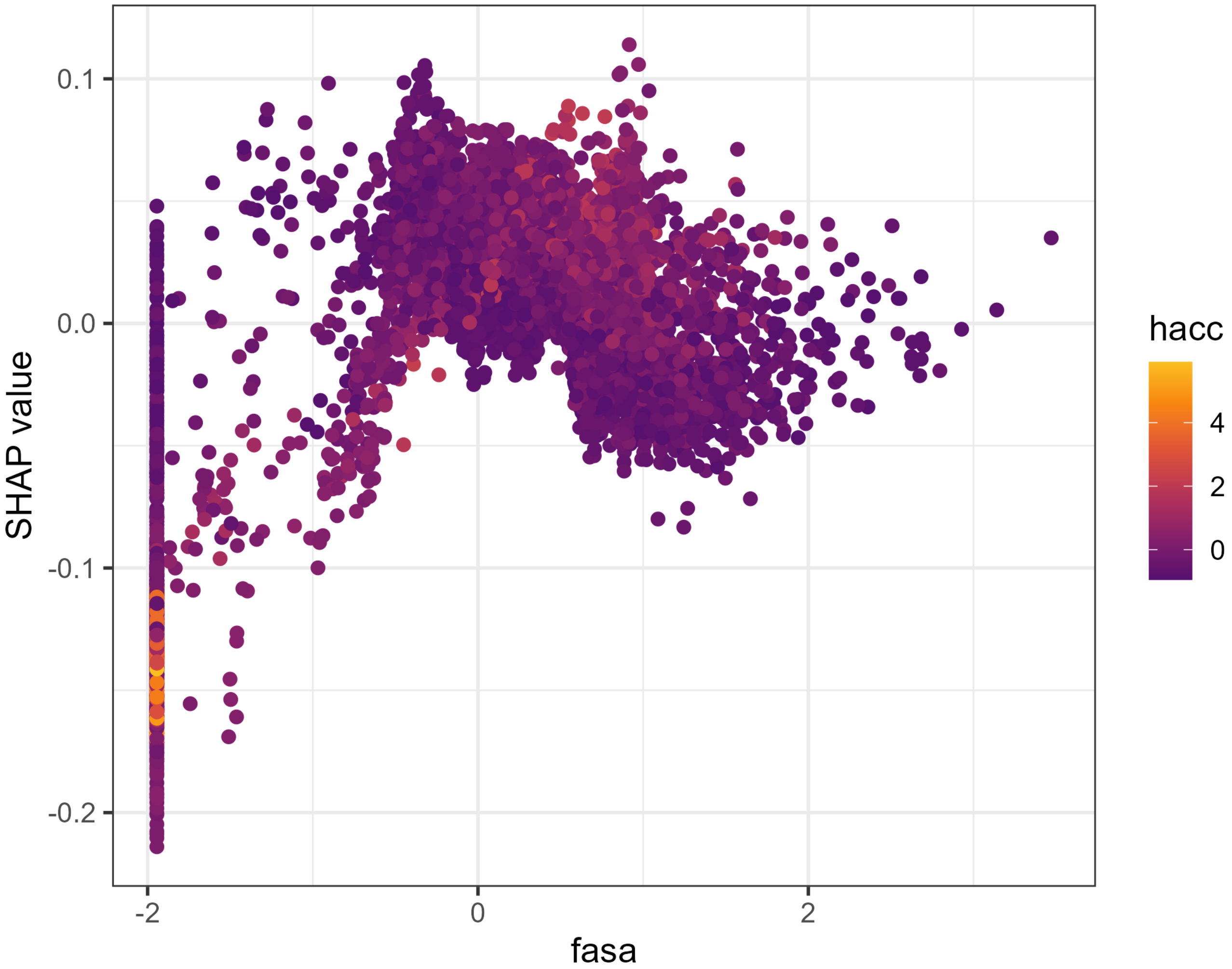

Figure S7. SHAP dependence plot for alogp

This figure shows the interval-specific contribution pattern of alogp in the deployed model.

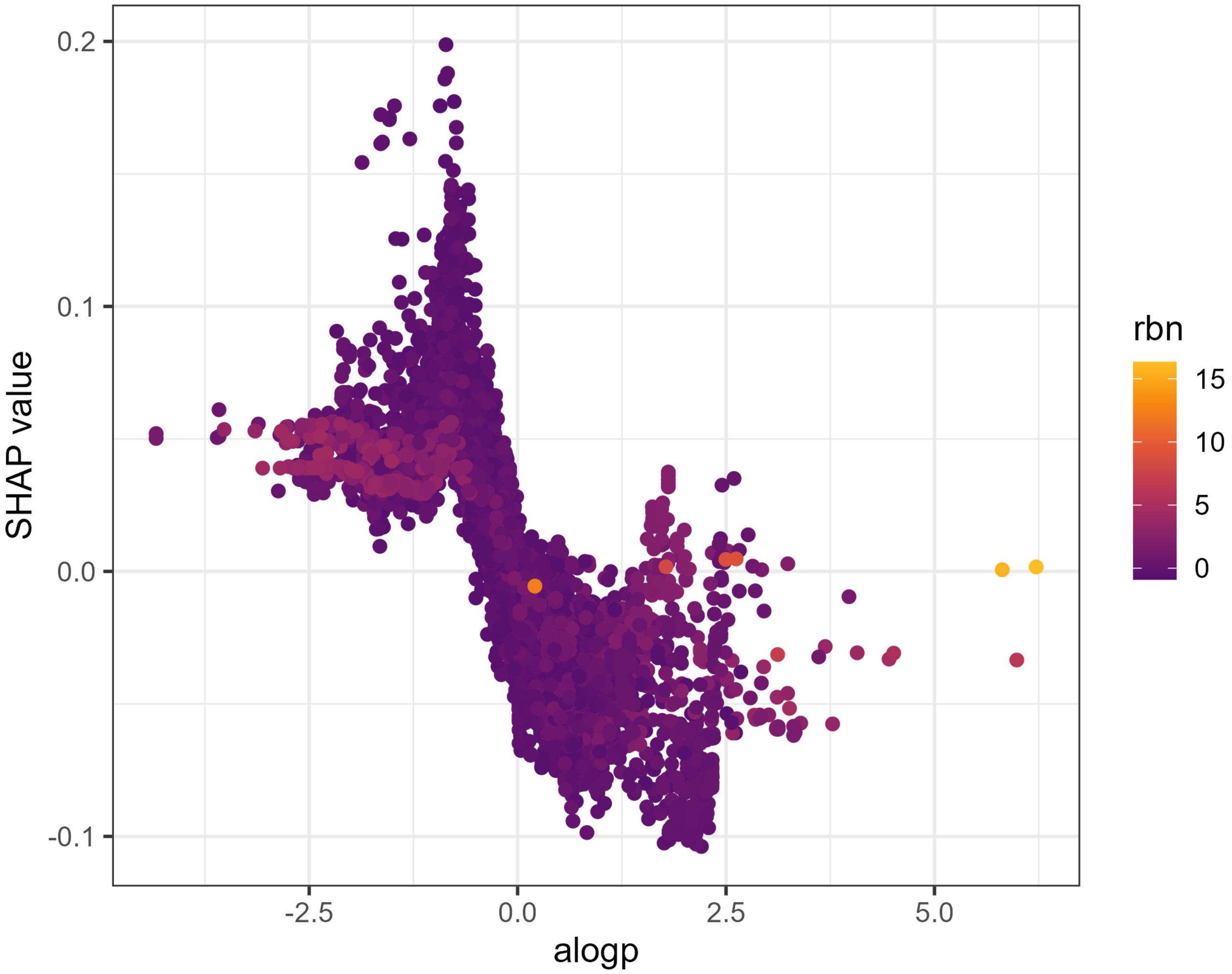

Figure S8. SHAP dependence plot for ob

This figure shows the contribution pattern of ob and complements Figures S5-S7 for descriptor-level interpretation.

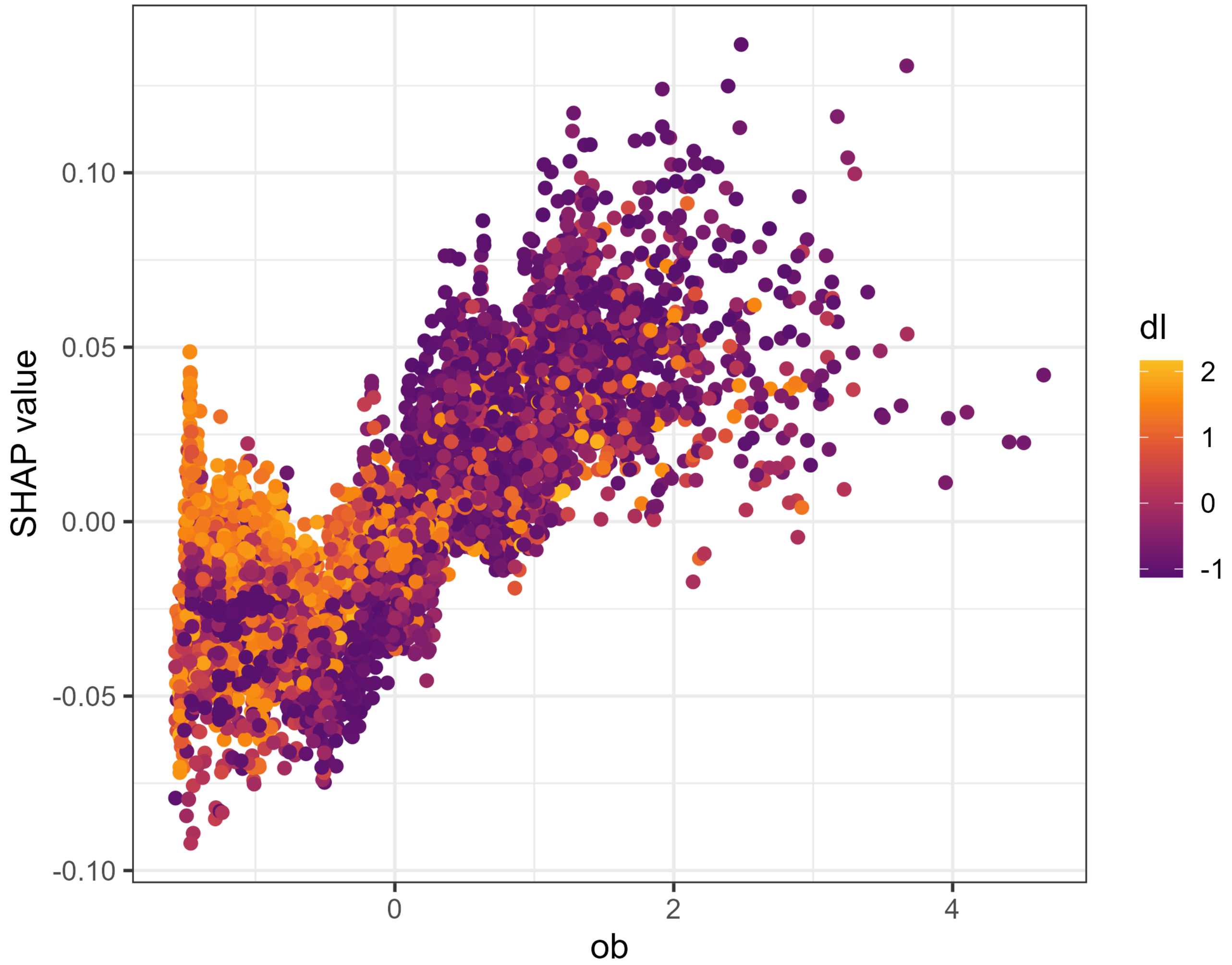

Figure S9. Heatmap of all 113 candidate model combinations

This figure extends the main-text candidate-model comparison by showing the full candidate set across the unified resampling framework.

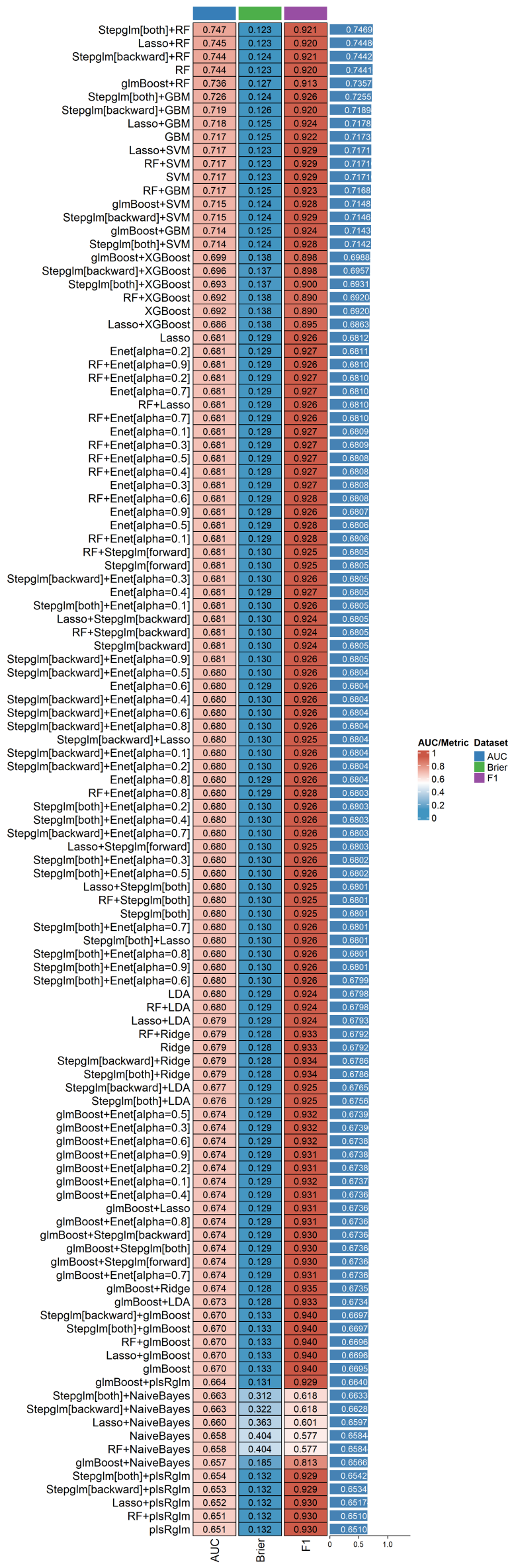

Figure S10. SHAP importance bar plot for the alternative Lasso+RF deployment

This figure is provided as a supplementary comparison to show the global explanatory structure of the alternative deployment retained for version tracing.

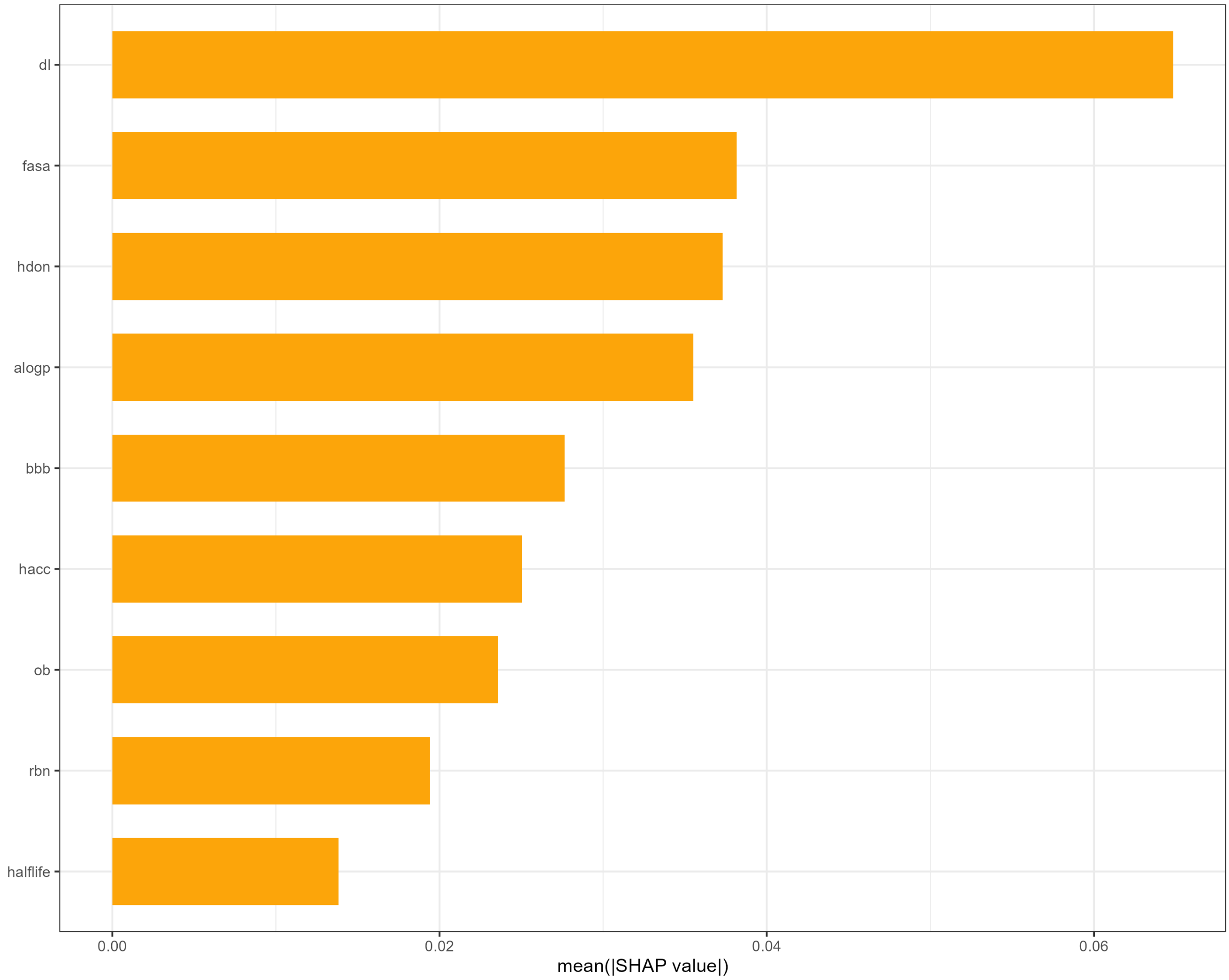

Figure S11. SHAP importance bar plot for the baseline RF deployment

This figure is provided as a supplementary comparison to show the explanatory structure of the baseline random forest deployment.

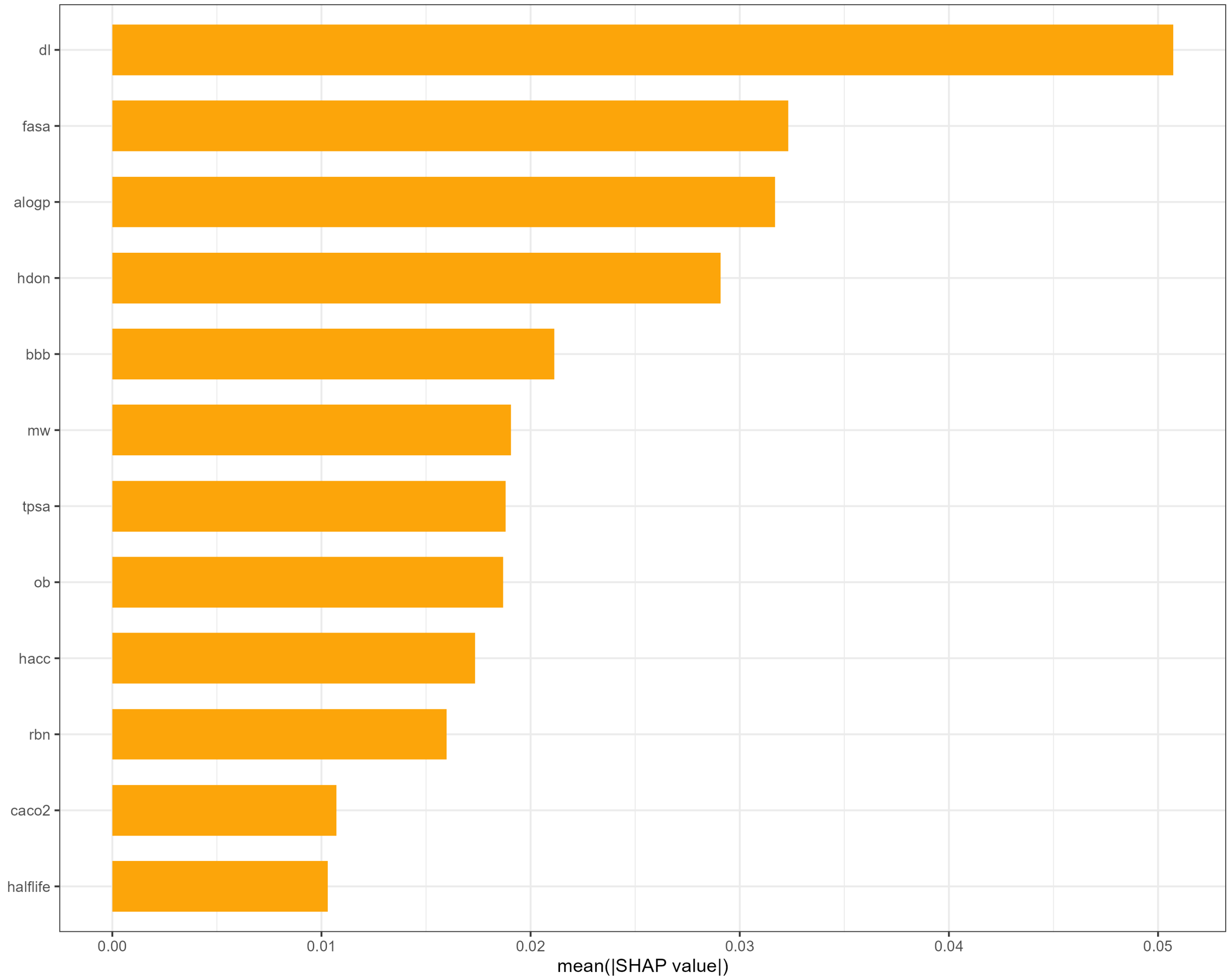

Figure S12. Out-of-fold PR curve of the deployed Stepglm[both]+RF model

This figure supplements the ROC-based performance display in the main text and presents the precision-recall behavior of the deployed model under the imbalanced classification setting.

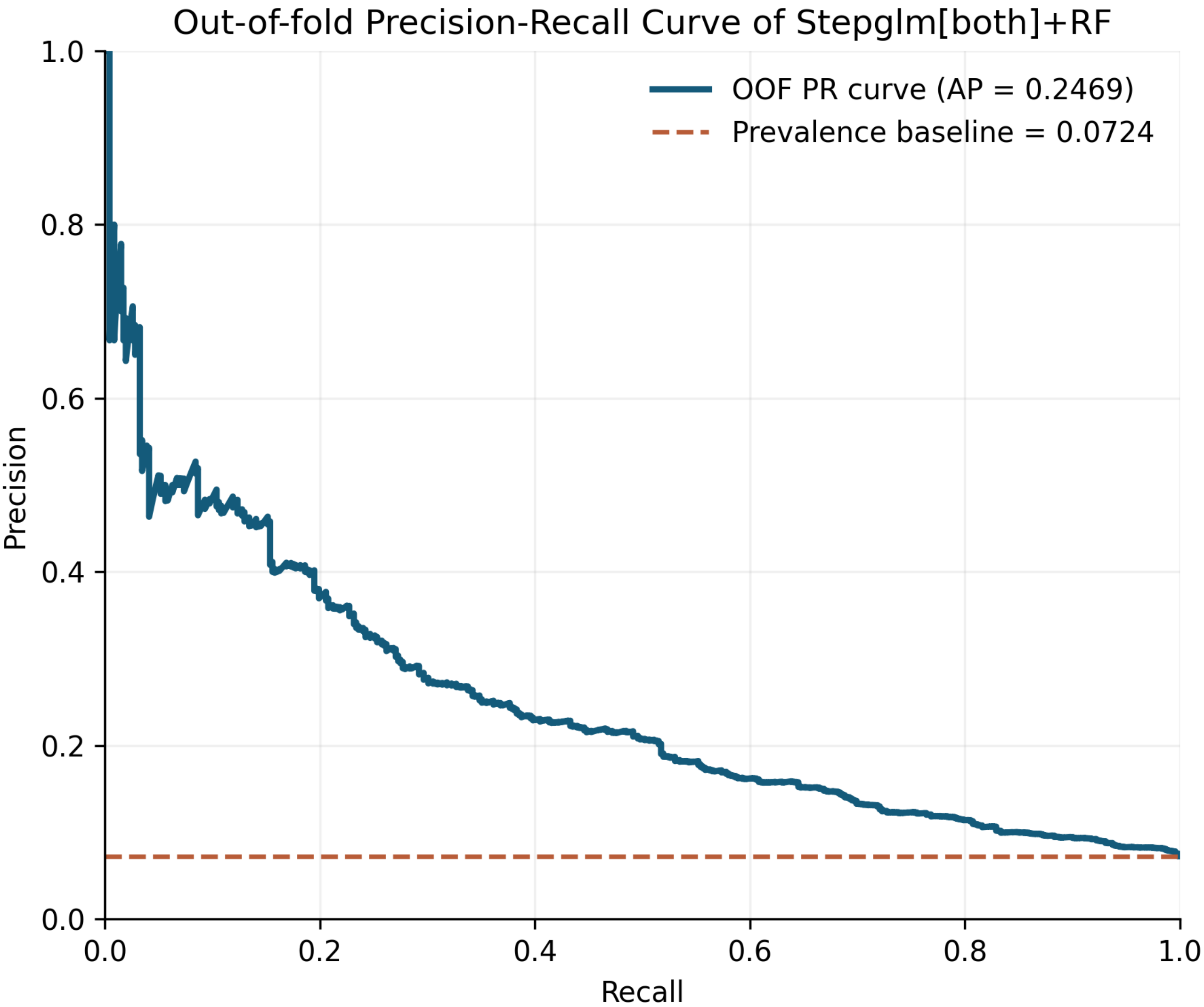

Supplementary Tables

Table S1. Cross-validation performance summary of all 113 candidate model combinations. The machine-readable table is provided separately as Additional file 2.

Table S2. Global SHAP feature importance values of the deployed model. The machine-readable table is provided separately as Additional file 3.

Table S3. Classification metrics at the selected operating cutoff. The machine-readable table is provided separately as Additional file 4.

Table S4. Multivariable logistic regression odds ratios for retained features. The machine-readable table is provided separately as Additional file 5.

Table S5. Archive file inventory and content description. The machine-readable table is provided separately as Additional file 6.

Table S6. Consistency summary of alternative deployed versions. The machine-readable table is provided separately as Additional file 7.

Table S7. PR-AUC summary of the top candidate models. The machine-readable table is provided separately as Additional file 8.

Supplementary notes on reproducibility

The supplementary machine-readable tables were retained in CSV format to facilitate editorial inspection, reviewer access, and downstream reuse. The frozen project package preserves the original input table, the preprocessed analysis matrix, cross-validation predictions, SHAP outputs, deployment objects, and archive descriptors referenced in the manuscript and in the supplementary files.
