# Supplementary material for: Shiny binary modeling platform: a reproducibility-oriented software platform for structured binary modeling with integrated interpretation, validation support, model comparison, and archive export
Source: Front Pharmacol. 2026 Sep 3;17:1905644. doi: 10.3389/fphar.2026.1905644 (PMC13582506; doi:10.3389/fphar.2026.1905644)
Supplement: Supplementary file 2 [file DataSheet11.zip › shiny binary modeling platform/CODE_AVAILABILITY.docx]

# Software Availability and Requirements

This file is formatted so that its contents can be adapted directly into a software/platform manuscript.

## Project name

Shiny Binary Modeling Platform

## Project home page

https://github.com/1303325800/shiny-binary-modeling-platform

## Archived version

https://github.com/1303325800/shiny-binary-modeling-platform/releases/tag/v0.1.0

## Operating system(s)

Primary development target: Windows desktop environment.

## Programming language

R

## Other requirements

Recent R 4.x installation and the packages listed in `DEPENDENCIES.md`.

## License

MIT License

## Restrictions to use by non-academics

No additional restrictions beyond the license terms, unless modified by the authors before release.

## Availability status for the present submission

The source code is publicly available through the GitHub repository and the tagged review release `v0.1.0`. A frozen local mirror of the same release is also included in the submission package as `Additional file 11.zip`.

## Recommended manuscript wording

`Project name: Shiny Binary Modeling Platform. Project home page: https://github.com/1303325800/shiny-binary-modeling-platform. Archived version: https://github.com/1303325800/shiny-binary-modeling-platform/releases/tag/v0.1.0. Operating system: Windows. Programming language: R. Other requirements: recent R 4.x installation and the packages listed in the repository dependency record. License: MIT. Restrictions to use by non-academics: none beyond the license terms. The source code is publicly available through the GitHub repository, the tagged review release v0.1.0, and the frozen local mirror provided as Additional file 11.zip.`
