# Supplementary material for: Shiny binary modeling platform: a reproducibility-oriented software platform for structured binary modeling with integrated interpretation, validation support, model comparison, and archive export
Source: Front Pharmacol. 2026 Sep 3;17:1905644. doi: 10.3389/fphar.2026.1905644 (PMC13582506; doi:10.3389/fphar.2026.1905644)
Supplement: Supplementary file 2 [file DataSheet11.zip › shiny binary modeling platform/DEPENDENCIES.docx]

# Dependency Record

This document summarizes the package-level dependencies identified from the current main application source file.

## Core web application framework

- shiny
- shinydashboard
- shinycssloaders
- DT
- shinyjqui

## Data import and wrangling

- dplyr
- tidyr
- readxl
- readr
- vroom
- janitor
- glue
- zip

## Modeling and evaluation

- tidymodels
- recipes
- rsample
- yardstick
- randomForestSRC
- glmnet
- plsRglm
- gbm
- mboost
- e1071
- MASS
- xgboost
- caret
- broom
- pROC
- nricens

## Explainability and interpretation

- kernelshap
- shapviz
- DALEX
- DALEXtra

## Visualization and reporting

- ggplot2
- patchwork
- ComplexHeatmap
- RColorBrewer
- rmarkdown
- gt
- scales
- forcats
- htmltools

## Base and utility namespaces used explicitly

- stats
- tools

## Notes

- This list is derived from the current application source and explicit namespace calls.
- The software is presently organized as a monolithic Shiny application file (`app.R`).
- A locked environment file such as `renv.lock` is recommended for a later public release.
