# Supplementary material for: Shiny binary modeling platform: a reproducibility-oriented software platform for structured binary modeling with integrated interpretation, validation support, model comparison, and archive export
Source: Front Pharmacol. 2026 Sep 3;17:1905644. doi: 10.3389/fphar.2026.1905644 (PMC13582506; doi:10.3389/fphar.2026.1905644)
Supplement: Supplementary file 2 [file DataSheet11.zip › shiny binary modeling platform/docs/ARCHITECTURE_AND_EXTENSIBILITY.docx]

# Architecture and Extensibility

## Scope

Shiny Binary Modeling Platform is a local R/Shiny application for structured binary modeling. The current implementation is organized as a single Shiny application file, `app.R`, with workflow modules implemented through Shiny server logic and shared reactive project state.

## Main workflow modules

| Module | Main role | Representative objects or outputs |

|---|---|---|

| Data setup and preprocessing | Upload a tabular dataset, define the target and predictors, clean names, map binary labels, and build the preprocessing recipe. | `raw_data`, `processed_data`, `recipe`, selected target and feature variables |

| Candidate-model evaluation | Run stratified cross-validation over fixed candidate model specifications. | CV metric summaries and fold-level predictions |

| Final model deployment | Refit a selected model specification and freeze the model object for downstream use. | Deployed model RDS, selected feature set, saved project object |

| SHAP interpretation | Generate global and local interpretation outputs when a compatible retained model is available. | SHAP matrix, feature-importance table, beeswarm/bar/waterfall outputs |

| External-validation support | Apply a frozen model and preprocessing recipe to a compatible user-supplied dataset. | Validation predictions, ROC/PR/calibration outputs when external data are supplied |

| Model comparison | Load two retained projects and compare their exported metrics and interpretation outputs. | Comparison metrics and comparison figures |

| Archive export | Export retained project outputs as a structured archive. | `ML_Project_Export` tables, figures, model summary, and optional standalone prediction app |

## Project object management

The application uses a shared Shiny `reactiveValues` object to retain the current analysis state. Important fields include `raw_data`, `processed_data`, `recipe`, `target_var`, `feature_vars`, `cv_results`, `cv_predictions`, `deployed_model_fit`, `deployed_model_name`, SHAP objects, validation outputs, comparison outputs, and `data_to_download`.

The full project save action writes an RDS list containing the deployed model, model name, preprocessing recipe, target variable, raw data, CV results, CV predictions, SHAP objects, and timestamp. The standalone prediction app export writes `model.rds`, `recipe.rds`, `background_data.rds`, and `raw_data_for_ui.rds` into a bundled Shiny calculator.

## Modeling engine and extension points

The modeling engine uses a unified dispatcher, `RunML()`, which calls learner-specific wrappers such as `RunEnet()`, `RunStepglm()`, `RunRF()`, `RunSVM()`, `RunGBM()`, `RunXGBoost()`, `RunNaiveBayes()`, `RunLDA()`, `RunglmBoost()`, and `RunplsRglm()`. To add a new learner, a developer should add a `RunNewMethod()` wrapper with the same input and return conventions, then register the method name in the candidate list used by the cross-validation loop.

## Current limitations

The present version is a monolithic Shiny application rather than a formal R package. The software is therefore easier to audit as a single-file app, but future public releases would benefit from formal package structure, automated tests, `renv.lock`, and a tested container image.
