# Supplementary material for: Shiny binary modeling platform: a reproducibility-oriented software platform for structured binary modeling with integrated interpretation, validation support, model comparison, and archive export
Source: Front Pharmacol. 2026 Sep 3;17:1905644. doi: 10.3389/fphar.2026.1905644 (PMC13582506; doi:10.3389/fphar.2026.1905644)
Supplement: Supplementary file 2 [file DataSheet11.zip › shiny binary modeling platform/docs/COMPARISON_WITH_STANDARD_R_WORKFLOW.docx]

# Comparison Draft: Standard R Script Workflow vs ToxXAI-TCM

This comparison is intentionally conservative. It describes workflow-level capabilities rather than claiming superiority in predictive performance.

| Dimension | Standard scattered R script workflow | ToxXAI-TCM |

|---|---|---|

| User interface | Usually command-line or notebook driven | Interactive Shiny dashboard |

| Data import and preprocessing entry point | Often split across separate scripts | Unified setup module inside one interface |

| Candidate-model benchmarking | Typically user-assembled from multiple scripts or notebooks | Integrated benchmarking within one application session |

| Final model freezing | Often manual and inconsistent across projects | Dedicated deployment step with frozen project/model export |

| Project object retention | Frequently partial or ad hoc | Save/load project objects through RDS-based workflow |

| Global explainability outputs | Usually requires separate code blocks | Built-in SHAP beeswarm, bar plot, export matrix |

| Local explainability outputs | Often custom scripting per model | Built-in prediction plus local SHAP waterfall/force plots |

| External validation workflow | Commonly handled in separate scripts | Dedicated validation module in the same platform |

| Model-to-model comparison | Often manual post-processing | Built-in comparison module with metric/statistics export |

| Archive export | Usually user-curated | One-click export of figures, tables, and archive bundle |

| Standalone app packaging | Rare in ordinary analysis scripts | Built-in export of a standalone prediction app bundle |

## Recommended manuscript use

Use this table to support a workflow/platform claim such as:

`Compared with a conventional scattered R script workflow, ToxXAI-TCM consolidates project setup, candidate-model screening, final deployment, interpretation, validation, comparison, and archive export into a single reproducible interface.`

Avoid overclaiming:

- Do not state that ToxXAI-TCM universally outperforms all conventional workflows.
- Do not use this table to imply superior biological validity.
- Keep the claim at the level of workflow integration, traceability, and practical reproducibility support.
