# Supplementary material for: Shiny binary modeling platform: a reproducibility-oriented software platform for structured binary modeling with integrated interpretation, validation support, model comparison, and archive export
Source: Front Pharmacol. 2026 Sep 3;17:1905644. doi: 10.3389/fphar.2026.1905644 (PMC13582506; doi:10.3389/fphar.2026.1905644)
Supplement: Supplementary file 2 [file DataSheet11.zip › shiny binary modeling platform/docs/EXAMPLE_WORKFLOW_AND_EXPECTED_OUTPUTS.docx]

# Example Workflow and Expected Outputs

## Example input

The manuscript demonstration uses the frozen compound-level input table preserved in the project archive:

`ML_Project_Export/Data_01_Raw_Input.csv`

Expected input summary:

- Rows: 6383
- Unique `mol_id` values: 6383
- Positive labels (`pure_toxic_label=1`): 462
- Negative labels (`pure_toxic_label=0`): 5921
- Modeling descriptors used in the manuscript demonstration: `ob`, `mw`, `alogp`, `caco2`, `bbb`, `halflife`, `hdon`, `hacc`, `dl`, `fasa`, `rbn`, and `tpsa`

## Reproduction workflow

1. Launch the app from the repository root with `shiny::runApp()`.
2. Upload the example input table.
3. Select `pure_toxic_label` as the target variable.
4. Define `1` as the positive/toxic class and `0` as the negative class.
5. Select the 12 ADME/physicochemical descriptors as predictors.
6. Run stratified 10-fold cross-validation with training-fold majority-class downsampling.
7. Export the project archive after model benchmarking and deployment.

## Expected file-level outputs

The full project archive is expected to contain the following core files when the corresponding workflow steps are completed:

| Output file | Expected role |

|---|---|

| `Data_01_Raw_Input.csv` | Uploaded raw input table |

| `Data_02_Preprocessed.csv` | Preprocessed modeling table |

| `Table_01_CV_Metrics_Summary.csv` | Candidate-model metric summary |

| `Data_03_CV_Raw_Predictions.csv` | Fold-level out-of-fold predictions |

| `Table_02_SHAP_Feature_Importance.csv` | Global SHAP feature-importance table when SHAP is run |

| `Data_04_SHAP_Global_Matrix.csv` | Global SHAP value matrix when SHAP is run |

| `Table_05_Final_Model_Features.csv` | Deployed model feature list |

| `Table_06_Optimal_Cutoff_Metrics.csv` | Cutoff summary |

| `Report_01_Model_Summary.txt` | Exported model summary |

## Expected audit values used in the revised manuscript

For the revised manuscript, the authoritative performance summaries are the revision audit files, not unreviewed historical labels in old figure filenames. Key values include:

- Stepglm[both]+RF merged OOF PR-AUC: 0.2469
- Stepglm[both]+RF merged OOF Brier score: 0.1230
- Stepglm[both]+RF toxic-class PPV/recall/F1/NPV at threshold 0.5: 0.2288 / 0.4091 / 0.2935 / 0.9509
- OOF prediction set: 6383 rows per model, preserving 462 positive and 5921 negative observations
- Calibration summary: mean predicted probability 0.2945 versus observed prevalence 0.0724; calibration intercept -1.8297; calibration slope 1.0997; 10-bin ECE 0.2221
