# Supplementary material for: Shiny binary modeling platform: a reproducibility-oriented software platform for structured binary modeling with integrated interpretation, validation support, model comparison, and archive export
Source: Front Pharmacol. 2026 Sep 3;17:1905644. doi: 10.3389/fphar.2026.1905644 (PMC13582506; doi:10.3389/fphar.2026.1905644)
Supplement: Supplementary file 2 [file DataSheet11.zip › shiny binary modeling platform/docs/EXTERNAL_VALIDATION_DATA_REQUIREMENTS.docx]

# External-validation dataset requirements

An external dataset is eligible for validating the frozen demonstration model only if it satisfies all of the following:

1. It was assembled independently of the model-development data.
2. It contains the same 12 predictor definitions and units, or a prespecified and reproducible mapping to them.
3. Its outcome provenance and positive-class definition are comparable with `pure_toxic_label`.
4. The frozen preprocessing recipe can be applied without refitting on the external outcomes.
5. Missingness, exclusions, class counts, and any distribution shift are reported before performance is examined.

An unrelated public binary-classification dataset, such as a clinical heart-disease dataset, cannot validate this frozen compound model because its variables, population, and outcome are incompatible.

For an eligible dataset, report ROC-AUC, PR-AUC, Brier score, calibration intercept and slope, confusion-matrix counts, PPV, sensitivity, F1, and NPV. Keep model parameters and the decision threshold frozen unless a separate recalibration analysis is explicitly labelled.
