# Supplementary material for: Shiny binary modeling platform: a reproducibility-oriented software platform for structured binary modeling with integrated interpretation, validation support, model comparison, and archive export
Source: Front Pharmacol. 2026 Sep 3;17:1905644. doi: 10.3389/fphar.2026.1905644 (PMC13582506; doi:10.3389/fphar.2026.1905644)
Supplement: Supplementary file 2 [file DataSheet11.zip › shiny binary modeling platform/docs/REPRODUCIBILITY_GUIDE.docx]

# Reproducibility Guide

## Tested scope

The review release is intended for a Windows 10/11 desktop R environment with a recent R 4.x installation. The current code package provides installation instructions, a dependency record, code availability metadata, architecture notes, and an example workflow checklist.

## Installation

1. Install R 4.x and optionally RStudio.
2. Open the repository root that contains `app.R`.
3. Install the packages listed in `DEPENDENCIES.md` or `INSTALL.md`.
4. Launch the app with:

shiny::runApp()

## Seed control

The cross-validation workflow uses `set.seed(123)` before creating folds. Fold-level majority-class downsampling uses `set.seed(123 + i)` inside the fold loop. The standalone prediction-app export samples SHAP background rows using `set.seed(123)`.

## Environment capture

Before final resubmission or public release, run the following commands in the R environment used to execute the app:

sessionInfo()

writeLines(capture.output(sessionInfo()), "sessionInfo_review_release.txt")

if (!requireNamespace("renv", quietly = TRUE)) install.packages("renv")

renv::init(bare = TRUE)

renv::snapshot(prompt = FALSE)

The original frozen review bundle did not include a generated `renv.lock`, and no tested Docker image is claimed for this revision. The current documentation therefore supports audit-friendly reproduction of the workflow and records the steps needed to create a fully locked environment on the author's R installation.

## Demonstration-data route

Use the frozen demonstration input table from the review archive, `ML_Project_Export/Data_01_Raw_Input.csv`, or the copied example file supplied with the revision audit materials. The expected data shape is 6383 records with 462 positive and 5921 negative labels.

## Expected outputs

The archive export should produce named files such as `Data_01_Raw_Input.csv`, `Data_02_Preprocessed.csv`, `Table_01_CV_Metrics_Summary.csv`, `Data_03_CV_Raw_Predictions.csv`, SHAP tables, selected-feature tables, cutoff summaries, and exported figures. The revised manuscript uses the audit files from the revision package for positive-class metrics, SHAP consistency, and PR-AUC/Brier/calibration summaries.
