# Supplementary material for: Shiny binary modeling platform: a reproducibility-oriented software platform for structured binary modeling with integrated interpretation, validation support, model comparison, and archive export
Source: Front Pharmacol. 2026 Sep 3;17:1905644. doi: 10.3389/fphar.2026.1905644 (PMC13582506; doi:10.3389/fphar.2026.1905644)
Supplement: Supplementary file 2 [file DataSheet11.zip › shiny binary modeling platform/docs/RUNTIME_MEMORY_BENCHMARK_PROTOCOL.docx]

# Runtime and memory benchmark protocol

Use this protocol for a new, reproducible run; do not estimate values from memory.

- Record CPU model/core count, RAM, operating system, R version, and `renv.lock` identifier.
- Record dataset dimensions, positive-class prevalence, descriptor count, candidate specifications, fold count, and random seeds.
- Run the complete benchmark three times after one unreported warm-up run.
- Record elapsed wall-clock time and peak resident memory for each run.
- Report mean, standard deviation, minimum, and maximum.
- State whether execution was sequential or parallel and report the number of workers.
- Do not claim superiority over another tool unless it is run on the same hardware, dataset, resampling splits, and comparable task settings.

Recommended manuscript wording after measurement:

> On [hardware and operating system], using R [version] and the locked package environment [identifier], the complete 113-specification, stratified 10-fold benchmark on 6383 records and 12 descriptors required a mean of [value] minutes (SD [value], range [value]-[value]; three measured runs after one warm-up) and reached a peak resident memory of [value] GB.
