# Supplementary material for: Shiny binary modeling platform: a reproducibility-oriented software platform for structured binary modeling with integrated interpretation, validation support, model comparison, and archive export
Source: Front Pharmacol. 2026 Sep 3;17:1905644. doi: 10.3389/fphar.2026.1905644 (PMC13582506; doi:10.3389/fphar.2026.1905644)
Supplement: Supplementary file 2 [file DataSheet11.zip › shiny binary modeling platform/docs/SHINYAPPS_IO_DEPLOYMENT.docx]

# Optional Web Demo Deployment Notes

This note is provided only as a preparation aid. The current repository has not been deployed from this environment.

## Minimal path to a hosted demo

1. Create an account on `shinyapps.io`.
2. Install deployment tooling in R:

install.packages("rsconnect")

3. Set account credentials in R using the token and secret from your shinyapps.io dashboard.
4. From the repository root, deploy:

rsconnect::deployApp(appDir = ".", appPrimaryDoc = "app.R")

## Review-period caution

If the manuscript states that code is available only for peer review at this stage, do not publish a public unrestricted demo before confirming the intended release policy.

## Safer alternative

If a public demo is not yet appropriate, provide:

- the frozen source archive,
- reviewer-only code access,
- and static screenshots in the manuscript or supplementary material.
