# Supplementary material for: Shiny binary modeling platform: a reproducibility-oriented software platform for structured binary modeling with integrated interpretation, validation support, model comparison, and archive export
Source: Front Pharmacol. 2026 Sep 3;17:1905644. doi: 10.3389/fphar.2026.1905644 (PMC13582506; doi:10.3389/fphar.2026.1905644)
Supplement: Supplementary file 2 [file DataSheet11.zip › shiny binary modeling platform/docs/TESTED_RUNTIME_AND_ENVIRONMENT_AUDIT.docx]

# Tested runtime and environment audit

## Test scope

- Operating system: Windows 11 x64 (build 26200)
- R: 4.5.2 (2025-10-31 ucrt), x86_64-w64-mingw32/x64
- Dataset: 6383 records, 462 positive and 5921 negative
- Predictors: 12 ADME/physicochemical descriptors
- Candidate specifications: 113
- Validation: stratified 10-fold cross-validation
- Downsampling: 1:1 majority-class downsampling within training folds only

## Measured result

- Complete run elapsed time: 518.509 seconds (8.64 minutes)
- Total model-fold fits attempted: 1130
- Successful model-fold fits: 1130
- Recorded errors: 0
- Observed peak R-process working set: approximately 2.08 GiB

This is one measured rerun on the author's computer, not a multi-machine scalability study. It supports local operability at the demonstrated workload but does not establish speed superiority over other software.

## Environment audit

- The submitted `app.R` was sourced successfully in a non-interactive smoke test.
- All 38 packages explicitly loaded by the application were available.
- Exact declared-package versions are recorded in `declared_dependency_inventory.csv`.
- Full R session information is recorded in `sessionInfo.txt`.
- A validated `renv.lock` was not produced because `renv` was not installed in the tested environment and package download was unavailable during the audit. The version inventory and session information are supplied as an environment record, but a clean-machine `renv::restore()` test remains future work.

## External validation

No independent dataset satisfying the frozen model's predictor and outcome-compatibility requirements was found in the available project materials. No unrelated binary-classification dataset was substituted, and no external-performance result is claimed.

The frozen Stepglm[both]+RF project was also subjected to a compatible-data prediction-interface smoke test. Its retained preprocessing recipe and model generated 6383 finite probabilities in the range 0 to 0.9599. This result verifies software execution only; because the records are not an independent external cohort, it is not reported as external-validation performance.
