# Supplementary material for: Shiny binary modeling platform: a reproducibility-oriented software platform for structured binary modeling with integrated interpretation, validation support, model comparison, and archive export
Source: Front Pharmacol. 2026 Sep 3;17:1905644. doi: 10.3389/fphar.2026.1905644 (PMC13582506; doi:10.3389/fphar.2026.1905644)
Supplement: Supplementary file 2 [file DataSheet11.zip › shiny binary modeling platform/INSTALL.docx]

# Installation and Local Launch

## Recommended environment

- Operating system: Windows 10/11 desktop environment
- R: recent R 4.x release
- RStudio: optional but recommended for local launching and package management

## Install required packages

Run the following command in R before launching the app:

```
required_pkgs <- c(
  "shiny", "shinydashboard", "shinycssloaders", "DT", "shinyjqui",
  "dplyr", "ggplot2", "patchwork", "janitor", "readxl", "vroom", "readr",
  "zip", "glue", "tidyr", "tidymodels", "recipes", "rsample", "yardstick",
  "kernelshap", "shapviz", "DALEX", "DALEXtra", "pROC", "openxlsx",
  "randomForestSRC", "glmnet", "plsRglm", "gbm", "mboost", "e1071",
  "MASS", "xgboost", "caret", "broom", "ComplexHeatmap", "RColorBrewer",
  "rmarkdown", "gt", "forcats", "htmltools", "scales", "nricens"
)

to_install <- setdiff(required_pkgs, rownames(installed.packages()))
if (length(to_install) > 0) install.packages(to_install)
```

Some machine-learning packages may prompt for additional system components depending on the local R setup.

## Launch the platform

From the repository root:

shiny::runApp()

If needed, you can also launch the app explicitly:

source("app.R")

## Suggested reproducibility capture

Before packaging a reviewer build, record:

sessionInfo()

and keep the output with the frozen submission bundle.

## Review-only distribution model

If the code is not being released publicly at the time of submission, provide the repository archive or frozen source bundle to editors/reviewers and state in the manuscript that public release will follow editorial decision.
