# Supplementary material for: Shiny binary modeling platform: a reproducibility-oriented software platform for structured binary modeling with integrated interpretation, validation support, model comparison, and archive export
Source: Front Pharmacol. 2026 Sep 3;17:1905644. doi: 10.3389/fphar.2026.1905644 (PMC13582506; doi:10.3389/fphar.2026.1905644)
Supplement: Supplementary file 2 [file DataSheet11.zip › shiny binary modeling platform/README.docx]

# Shiny Binary Modeling Platform

This repository contains a Shiny-based desktop platform for structured binary modeling. The current implementation integrates data import, preprocessing, candidate-model benchmarking, final model deployment, SHAP-based interpretation, external validation, model-to-model comparison, and archive export into a single interface.

The source code is publicly available through this GitHub repository and the tagged release `v0.1.0`, which is the frozen review version referenced in the accompanying software manuscript.

## What the platform currently supports

- Structured binary classification workflows in a Shiny dashboard.
- Import of tabular datasets and preprocessing for downstream modeling.
- Candidate-model evaluation across multiple machine-learning algorithms.
- Deployment of a selected final model on the full dataset.
- Global and local SHAP outputs for model interpretation.
- External validation of a deployed model on an independent dataset.
- Side-by-side comparison of two frozen model projects.
- Export of project archives, figures, metrics tables, and app bundles.
- Export of a standalone prediction Shiny calculator containing the frozen model.

## Repository contents

- `app.R`: main Shiny application.
- `INSTALL.md`: local installation and launch instructions.
- `DEPENDENCIES.md`: package-level dependency record.
- `CODE_AVAILABILITY.md`: manuscript-ready software availability statement.
- `docs/COMPARISON_WITH_STANDARD_R_WORKFLOW.md`: conservative feature comparison against a conventional script-based workflow.
- `docs/SHINYAPPS_IO_DEPLOYMENT.md`: optional notes for preparing a web demo deployment.
- `docs/ARCHITECTURE_AND_EXTENSIBILITY.md`: module-level architecture, retained project-object structure, and extension points.
- `docs/REPRODUCIBILITY_GUIDE.md`: installation, seed-control, environment-capture, and reproducibility-status notes.
- `docs/EXAMPLE_WORKFLOW_AND_EXPECTED_OUTPUTS.md`: demonstration-data route and expected output checklist.

## Quick start

1. Install a recent R 4.x environment on Windows.
2. Install the packages listed in `INSTALL.md`.
3. Open an R session in this repository folder.
4. Run:

shiny::runApp()

If you keep the application file name as `app.R`, Shiny will launch the platform automatically from the repository root.

## Suggested GitHub repository setup

Recommended repository name:

`shiny-binary-modeling-platform`

Recommended short description:

`A reproducible Shiny platform for structured binary modeling with integrated interpretation, validation, model comparison, and archive export`

Recommended topics:

`shiny`, `r`, `machine-learning`, `binary-classification`, `shap`, `reproducible-research`, `workflow`

## Availability note for the manuscript

Current intended statement:

`The source code of the platform is publicly available through the GitHub repository and the tagged review release v0.1.0.`

## Scope note

The current manuscript should treat the 6383-compound dataset as a platform demonstration use case rather than as the sole basis for broad scientific generalization.
